# Roles and Programming of Arabidopsis ARGONAUTE Proteins during *Turnip Mosaic Virus* Infection

**DOI:** 10.1371/journal.ppat.1004755

**Published:** 2015-03-25

**Authors:** Hernan Garcia-Ruiz, Alberto Carbonell, J. Steen Hoyer, Noah Fahlgren, Kerrigan B. Gilbert, Atsushi Takeda, Annalisa Giampetruzzi, Mayra T. Garcia Ruiz, Michaela G. McGinn, Nicholas Lowery, Maria T. Martinez Baladejo, James C. Carrington

**Affiliations:** 1 Donald Danforth Plant Science Center, St. Louis, Missouri, United States of America; 2 Center for Genome Research and Biocomputing, Department of Botany and Plant Pathology, Oregon State University, Corvallis, Oregon, United States of America; 3 Computational and Systems Biology Program, Washington University in St. Louis, St. Louis, Missouri, United States of America; The Ohio State University, UNITED STATES

## Abstract

In eukaryotes, ARGONAUTE proteins (AGOs) associate with microRNAs (miRNAs), short interfering RNAs (siRNAs), and other classes of small RNAs to regulate target RNA or target loci. Viral infection in plants induces a potent and highly specific antiviral RNA silencing response characterized by the formation of virus-derived siRNAs. *Arabidopsis thaliana* has ten *AGO* genes of which *AGO1*, *AGO2*, and *AGO7* have been shown to play roles in antiviral defense. A genetic analysis was used to identify and characterize the roles of AGO proteins in antiviral defense against *Turnip mosaic virus* (TuMV) in Arabidopsis. AGO1, AGO2 and AGO10 promoted anti-TuMV defense in a modular way in various organs, with AGO2 providing a prominent antiviral role in leaves. AGO5, AGO7 and AGO10 had minor effects in leaves. AGO1 and AGO10 had overlapping antiviral functions in inflorescence tissues after systemic movement of the virus, although the roles of AGO1 and AGO10 accounted for only a minor amount of the overall antiviral activity. By combining AGO protein immunoprecipitation with high-throughput sequencing of associated small RNAs, AGO2, AGO10, and to a lesser extent AGO1 were shown to associate with siRNAs derived from silencing suppressor (HC-Pro)-deficient TuMV-AS9, but not with siRNAs derived from wild-type TuMV. Co-immunoprecipitation and small RNA sequencing revealed that viral siRNAs broadly associated with wild-type HC-Pro during TuMV infection. These results support the hypothesis that suppression of antiviral silencing during TuMV infection, at least in part, occurs through sequestration of virus-derived siRNAs away from antiviral AGO proteins by HC-Pro. These findings indicate that distinct AGO proteins function as antiviral modules, and provide a molecular explanation for the silencing suppressor activity of HC-Pro.

## Author Summary

RNA silencing is a primary, adaptive defense system against viruses in plants. Viruses have evolved counter-defensive mechanisms that inhibit RNA silencing through the activity of silencing suppressor proteins. Understanding how antiviral silencing is controlled, and how suppressor proteins function, is essential for understanding how plants normally resist viruses, why some viruses are highly virulent in different hosts, and how sustainable antiviral resistance strategies can be deployed in agricultural settings. We used a mutant version of *Turnip mosaic virus* lacking a functional silencing suppressor (HC-Pro) to understand the genetic requirements for resistance in the model plant *Arabidopsis thaliana*. We focused on ARGONAUTE proteins, which have long been hypothesized to bind short interfering RNAs (siRNAs) derived from virus genomes for use as sequence-specific guides to recognize and target viral RNA for degradation or repression. We demonstrated specialized antiviral roles for specific ARGONAUTES and showed that several can bind viral siRNAs from across the entire viral genome. However, ARGONAUTE proteins are only loaded with virus-derived siRNAs in the absence of HC-Pro, which we showed binds siRNAs from the viral genome. This indicates that several AGO proteins, which collectively are necessary for full anti-TuMV defense, need to properly load virus-derived siRNAs to execute their antiviral roles.

## Introduction

In plants, RNA silencing is a highly specific and adaptive defense mechanism against viruses [1, 2]. Factors involved in antiviral silencing overlap with those of endogenous small RNA pathways, and include i) small RNA biogenesis components such as Dicer-like ribonucleases (DCLs), RNA-dependent RNA polymerases (RDRs), and double-stranded RNA (dsRNA) binding proteins, and ii) ARGONAUTE (AGO) proteins, which function as small RNA-binding effectors [3–6].

RNA-based silencing is triggered by dsRNA that is processed by DCLs into 21- to 24-nt short interfering RNAs (siRNAs), which subsequently associated with AGO proteins to form the RNA-induced silencing complex (RISC) [7, 8]. Inhibition of target RNA can occur by endonucleolytic cleavage (“slicing”), translational repression, or delivery of chromatin-modifying complexes to a locus [9–11, 12]. In some cases, amplification of the silencing response occurs by triggering dsRNA synthesis and secondary siRNA accumulation [13].

Viruses are inducers of RNA silencing; infected plants accumulate large amounts of siRNAs derived from viral RNAs [1]. Most plant viruses encode one or more silencing suppressor proteins that interfere with antiviral RNA silencing [13, 14]. One mechanism of silencing suppression by viral suppressors is through sequestration of siRNA duplexes [1], preventing assembly of the RISC effector complex. Other viral silencing suppressors promote AGO degradation [15–19], prevent slicing or degradation of target RNAs by associating with AGOs [20, 21], or use other mechanisms (for a recent review see Nakahara and Masuta 2014 [22]). In effect, viral suppressors mask the effects of antiviral silencing, making genetic analysis of antiviral silencing factors in host plants dependent on the use of suppressor-deficient viruses [3, 4, 6, 23].

*A*. *thaliana* has ten *AGO* genes [24], of which *AGO1*, *AGO2* and *AGO7* have been implicated in antiviral defense against various viruses by genetic and biochemical criteria [6, 25–31]. Antiviral roles for AGO3 and AGO5 have also been suggested based on virus-derived siRNA association and/or *in vitro* analyses [8, 32]. One model for AGO antiviral activity states that AGO proteins bind virus-derived siRNAs and directly repress viral RNA through slicing, translational repression, or other mechanisms [2, 8, 33]. Given that AGO-dependent regulation of gene expression affects numerous biological processes, including DNA repair [34], AGO proteins might also affect virus replication indirectly through regulation of genes with roles in defense. For example, AGO2-miR393* complexes regulate the expression of *MEMBRIN 12 (MEMB12)*, which is required for resistance to *Pseudomonas syringae* in *A*. *thaliana* [35]. Moreover, some AGO proteins are known to modulate the activity of other AGO proteins [36, 37], which could affect AGOs with roles in antiviral defense.

Potyviral HC-Pro is a suppressor of RNA silencing. As shown using potyviruses like *Turnip mosaic virus* (TuMV) [23, 38], the counter-defensive function of HC-Pro is necessary for establishment of infection or systemic spread. HC-Pro has been proposed to function through sequestration of virus-derived siRNAs [39–44]. HC-Pro may also function through physical interaction with factors like the transcription factor RAV2 [45], translation initiation factors eIF(iso)4E and eIF4E [46], calmodulin-related protein (CaM) [47], auxiliary proteins like Heat Shock Protein 90 (HSP90) [48], and/or through effects on downstream defense or silencing factors [49, 50]. Here, the role of several *A*. *thaliana* AGOs in antiviral defense against TuMV was analyzed in various organs of systemically infected plants. The impact of HC-Pro on the loading of antiviral AGOs with virus-derived siRNAs was also studied.

## Results

### AGO2 has a strong antiviral effect in leaves

**Fig 1:**
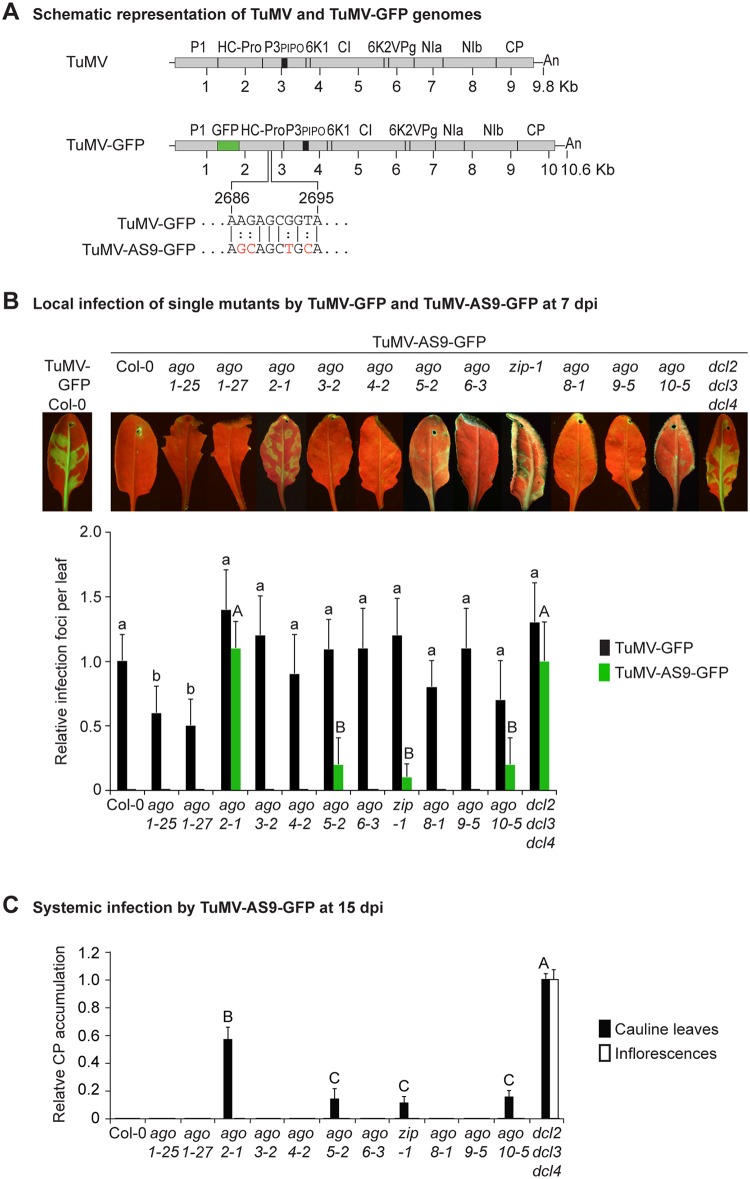
Local and systemic infection of *A*. *thaliana* single *ago* mutants by TuMV-GFP and TuMV-AS9-GFP. (A) Schematic representation of the TuMV and TuMV-GFP genomes showing insertion of GFP between P1 and HC-Pro, and the AS9 mutation on HC-Pro. (B) Visualization of local infection of inoculated rosette leaves. Pictures were taken at 7 days post inoculation (dpi). Col-0 infected by TuMV-GFP is shown for comparison. The histogram shows average (+ SE) infection efficiency of 14 plants, each with four inoculated leaves. Infection efficiency by TuMV-GFP or TuMV-AS9-GFP is expressed relative to Col-0 (9.8 ± 2 foci per leaf) or to *dcl2–1 dcl3–1 dcl4–2* (3.5 ± 1.4 foci per leaf), respectively. For each virus, bars with the same letter are not statistically different (Tukey’s test with α = 0.05). (C) TuMV-AS9-GFP coat protein (CP) accumulation in noninoculated cauline leaves and in inflorescence at 15 dpi is determined by immunoblotting and expressed relative to *dcl2–1 dcl3–1 dcl4–2*. The histogram shows average (+ SE) of four biological replicates. Bars with the same letter are not statistically different (Tukey’s test with α = 0.05). The experiment was repeated twice with similar results.

Three of the ten *A*. *thaliana AGO* genes have been implicated in antiviral defense: AGO1 against *Cucumber mosaic virus* (CMV) [25], *Turnip crinkle virus* (TCV) [6, 33], and *Brome mosaic virus* (BMV) [30]; AGO2 against TCV [26], *Potato virus X* (PVX) [27], CMV [26, 28, 29], and TuMV [31]; and AGO7 against TCV [6]. To identify the complete set of AGOs required for antiviral defense against TuMV in *A*. *thaliana*, single, double, and triple *ago* mutants were inoculated with a GFP-expressing form of parental TuMV (TuMV-GFP) and HC-Pro-deficient TuMV-AS9-GFP [23]. The GFP sequence was inserted between P1 and HC-Pro sequences (Fig. 1A). Both TuMV and TuMV-GFP require translation factor eIF(iso)4E [51], and lead to similar virus-derived siRNA profiles in wild-type and *dicer-like* mutant *A*. *thaliana* [23]. To determine if AGOs have spatially distinct functions, TuMV-GFP and TuMV-AS9-GFP accumulation was analyzed in inoculated rosette leaves, and in noninoculated cauline leaves and inflorescences. Establishment of local and systemic infection was monitored using GFP fluorescence, and virus accumulation in inoculated and noninoculated tissues was measured by immunoblotting assays (coat protein) as described [23].

Parental TuMV-GFP was detected in inoculated leaves and noninoculated inflorescences of all single *ago* mutants analyzed (Table 1 and Fig. 1B). Local infection of single *ago1* mutants was significantly lower than that of wild-type Col-0 (Fig. 1B), but this was likely due to the difficulty of inoculating the smaller leaves of hypomorphic mutants containing *ago1* alleles.

**Table 1:** TuMV-GFP and TuMV-AS9-GFP infection in single *ago* mutants ^a^.

Virus	Arabidopsis genotype	Plants inoculated	Local infection	Cauline leaves	Inflorescence
TuMV-GFP					
	Col-0	14	14	14	14
	*ago1–25*	14	14	14	14
	*ago1–27*	14	14	14	14
	*ago2–1*	14	14	14	14
	*ago3–2*	14	14	14	14
	*ago4–2*	14	14	14	14
	*ago5–2*	14	14	14	14
	*ago6–3*	14	14	14	14
	*zip-1*	14	14	14	14
	*ago8–1*	14	14	14	14
	*ago9–5*	14	14	14	14
	*ago10–5*	14	14	14	14
	*dcl2–1 dcl3–1 dcl4–2*	14	14	14	14
TuMV-AS9-GFP					
	Col-0	14	0	0	0
	*ago1–25*	14	0	0	0
	*ago1–27*	14	0	0	0
	*ago2–1*	14	14	14	0
	*ago3–2*	14	0	0	0
	*ago4–2*	14	0	0	0
	*ago5–2*	14	6	6	0
	*ago6–3*	14	0	0	0
	*zip-1*	14	5	5	0
	*ago8–1*	14	0	0	0
	*ago9–5*	14	0	0	0
	*ago10–5*	14	7	7	0
	*dcl2–1 dcl3–1 dcl4–2*	14	14	14	14

^a^ Number of plants showing local and systemic infections were scored by GFP fluorescence under UV illumination. Local infection foci were counted at 7 days post-inoculation (dpi). All other data is from plants at 15 dpi.

As described for *A*. *thaliana rdr* and *dcl* mutants [23], suppressor-deficient TuMV-AS9-GFP was expected to infect only those plants lacking one or more AGOs with a role in antiviral defense. No infection foci were detected in wild-type Col-0 plants (Fig. 1B and Table 1). Local infection foci of suppressor-deficient TuMV-AS9-GFP were readily visible at 7 days post inoculation (dpi) in *ago2–1* mutant plants (Fig. 1B and Table 1), and infection efficiency was not significantly different than that of the *dcl2–1 dcl3–1 dcl4–2* triple mutant, which served as the hypersusceptible, silencing-deficient control (Fig. 1B) [23]. Low numbers of infection foci were also detected in single *ago5–2*, *zip-1* (*ago7*), and *ago10–5* mutant plants (Fig. 1B and Table 1). Systemic movement of TuMV-AS9-GFP into cauline leaves was detected at 15 dpi in *ago2–1* plants, and also in *ago5–2*, *zip-1*, and *ago10–5* plants though at significantly lower levels (Fig. 1C and Table 1). In cauline leaves from single *ago2–1* mutant plants, TuMV-AS9-GFP accumulated to approximately 60% of the level measured in *dcl2–1 dcl3–1 dcl4–2* plants, while *ago5–2*, *zip-1*, and *ago10–5* plants accumulated TuMV-AS9-GFP to approximately 10% of the levels measured in the hypersusceptible control (Fig. 1C). In contrast to *dcl2–1 dcl3–1 dcl4–2* plants, systemic infection by TuMV-AS9-GFP did not reach inflorescence tissues in any of the single *ago* mutant or Col-0 plants (Fig. 1C and Table 1). Systemic infection did not reach cauline leaves in any of the other single *ago* mutants or Col-0 plants (Fig. 1C and Table 1).

### AGO1 and AGO10 have modest antiviral effects in inflorescences

**Fig 2:**
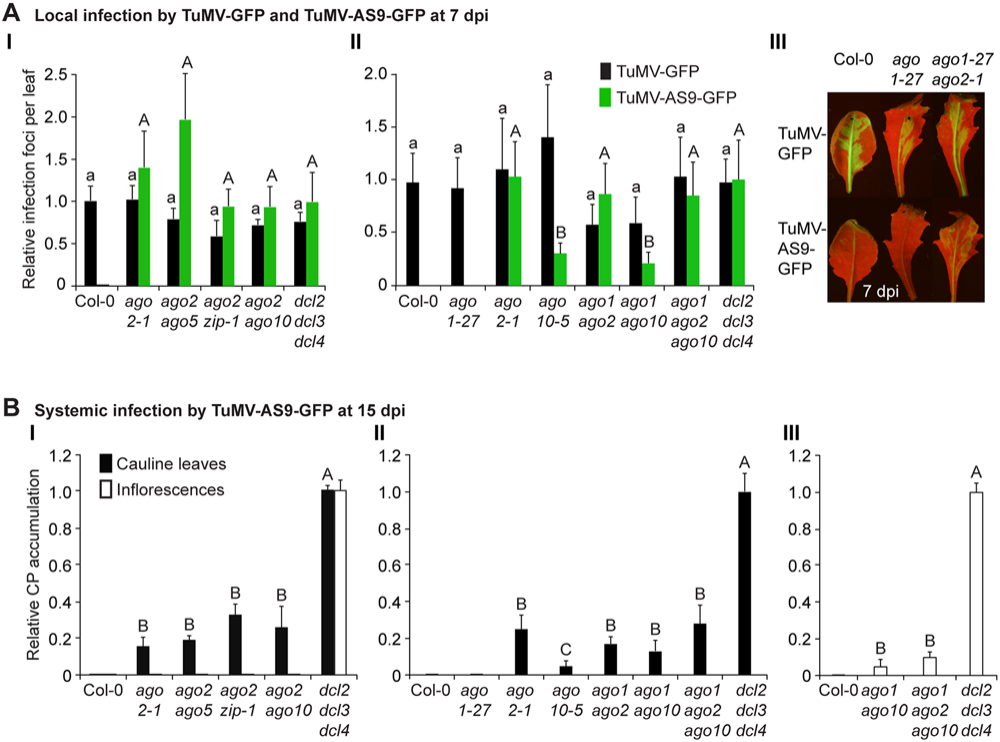
Local and systemic infection of a selected group of double and triple *ago* mutants by TuMV-GFP and TuMV-AS9-GFP. (A) Local infection efficiency. Panel I: infection efficiency of TuMV-GFP or TuMV-AS9-GFP is expressed relative to Col-0 (19.6 ± 3.3 foci per leaf) or to *dcl2–1 dcl3–1 dcl4–2* (2.2 ± 0.7 foci per leaf), respectively. The histogram shows the average (+ SE) of 10 plants, each with four inoculated leaves. Panel II: local infection of inoculated rosette leaves for a selected group of mutants harboring *ago1–27*. The histogram shows average (+ SE) infection efficiency of 14 plants, each with four inoculated leaves. Infection efficiency of TuMV-GFP or TuMV-AS9-GFP is expressed relative to Col-0 (4.1 ± 1.2 foci per leaf) or to *dcl2–1 dcl3–1 dcl4–2* (2.8 ± 1.1 foci per leaf), respectively. Panel III: Representative leaves of *ago1–27* single and *ago1–27 ago2–1* double mutants showing TuMV-GFP local infection foci. *ago1–27 ago2–1*, but not *ago1–27*, was infected by TuMV-AS9-GFP. Col-0 is shown for comparison. Pictures were taken at 7 dpi under UV light. (B) Systemic infection. TuMV-AS9-GFP coat protein accumulation in noninoculated cauline leaves and in inflorescence at 15 dpi. Panel I: double mutants harboring *ago2–1*. Panel II: double and triple mutants harboring *ago1–27* and *ago10–5*. The histograms show average (+ SE) of four biological replicates, expressed relative to *dcl2–1 dcl3–1 dcl4–2*. Bars with the same letter are not statistically different (Tukey’s test with α = 0.05). Panel III: in double and triple mutants harboring *ago1–27*, inflorescence samples were collected only from clusters showing systemic GFP.

To determine if the major effect of AGO2 was additive with the minor effects of AGO5, AGO7 and AGO10, and to examine if AGO1 possessed redundant or masked activities, double and triple *ago* mutant plants were inoculated with TuMV-GFP or TuMV-AS9-GFP, and virus accumulation was measured in inoculated and noninoculated organs as described above. To reduce the effect of differences in leaf size, we planted mutant lines with the *ago1–27* allele one week earlier than the other mutant lines inoculated at the same time. Parental TuMV-GFP infected locally (Fig. 2A panels I and II) and moved systemically into the inflorescence of all double and triple *ago* mutants analyzed (Tables 2 and 3), with no significant differences in infection efficiency.

**Table 2:** TuMV-GFP and TuMV-AS9-GFP infection in selected *ago2–1* based double mutants^a^.

Virus	Arabidopsis	Plants	Local	Cauline	Inflorescence
	genotype	inoculated	infection	leaves	
TuMV-GFP					
	Col-0	10	10	10	10
	*ago2–1*	10	10	10	10
	*ago2–1 ago5–2*	10	10	10	10
	*ago2–1 zip-1*	10	10	10	10
	*ago2–1 ago10–5*	10	10	10	10
	*dcl2–1 dcl3–1 dcl4–2*	10	10	10	10
TuMV-AS9-GFP					
	Col-0	10	0	0	0
	*ago2–1*	10	10	10	0
	*ago2–1 ago5–2*	10	10	10	0
	*ago2–1 zip-1*	10	10	10	0
	*ago2–1 ago10–5*	10	10	10	0
	*dcl2–1 dcl3–1 dcl4–2*	10	10	10	10

^a^ Number of plants showing local and systemic infections were scored by GFP fluorescence under UV illumination. Local infection foci were counted at 7 days post-inoculation (dpi). All other data is from plants at 15 dpi.

**Table 3:** TuMV-GFP and TuMV-AS9-GFP infection in selected ***ago1–27*** based combination mutants^a^.

Virus	Arabidopsis	Plants	Local	Cauline	Inflorescence	Percent ^b^
	genotype	inoculated	infection	leaves		
TuMV-GFP						
	Col-0	14	14	14	14	100
	*ago1–27*	14	14	14	14	100
	*ago2–1*	14	14	14	14	100
	*ago10–5*	14	14	14	14	100
	*ago1–27 ago2–1*	14	14	14	14	100
	*ago1–27 ago10–5*	14	14	14	14	100
	*ago1–27 ago2–1 ago10–5*	14	14	14	14	100
	*dcl2–1 dcl3–1 dcl4–2*	14	14	14	14	100
TuMV-AS9-GFP						
	Col-0	14	0	0	0	0
	*ago1–27*	14	0	0	0	0
	*ago2–1*	14	14	14	0	0
	*ago10–5*	14	8	6	0	0
	*ago1–27 ago2–1*	14	14	14	0	0
	*ago1–27 ago10–5*	14	14	14	3	4 ±1
	*ago1–27 ago2–1 ago10–5*	14	14	14	8	14 ±2
	*dcl2–1 dcl3–1 dcl4–2*	14	14	14	14	100

^a^ Number of plants showing local and systemic infections were scored by GFP fluorescence under UV illumination. Local infection foci were counted at 7 days post-inoculation. All other data is from plants at 15 dpi.

^b^ Proportion (%) of inflorescence clusters showing GFP with respect to the total number of clusters on each plant with inflorescence GFP fluorescence.

In double mutants harboring the *ago2–1* allele and one of *ago5–2*, *zip-1*, or *ago10–5* alleles, no significant differences in number of infection foci were detected at 7 dpi in rosette leaves inoculated with TuMV-AS9-GFP (Fig. 2A panel I and Table 2). Similarly, no significant differences were detected in TuMV-AS9-GFP coat protein accumulation in cauline leaves at 15 dpi (Fig. 2B panel I). As observed for the *ago* single mutants, TuMV-AS9-GFP was not detected in inflorescences from double mutant plants containing the *ago2–1* allele (Fig. 2B panel I). These results indicate that the minor activities of AGO5, AGO7 and AGO10 are not additive with the major antiviral activity of AGO2. Double and triple mutants harboring the *ago1–27* allele were generated and inoculated with parental TuMV-GFP or suppressor-deficient TuMV-AS9-GFP. Col-0 plants and *ago1–27*, *ago2–1* and *ago10–5* single mutant lines were included as controls. Local TuMV-AS9-GFP infection foci were observed in inoculated rosette leaves, and virus was detected in noninoculated cauline leaves, from *ago1–27 ago2–1* double mutant plants, but *ago1–27* had no enhancing or suppressing effects when combined with *ago2–1* (panel II in Fig. 2A and 2B, Table 3). Combining *ago1–27* with *ago10–5*, or with *ago2–1* and *ago10–5* in a triple mutant, had no effects on local TuMV-AS9-GFP infection foci (Fig. 2A panel II) or accumulation in cauline leaves beyond those measured in the single *ago2* or double *ago2 ago10* mutants (Fig. 2B panels I and II, and Table 3). However, combining *ago1–27* with *ago10–5* resulted in an increase in TuMV-AS9-GFP CP accumulation in cauline leaves relative to single *ago10–5* mutants (Fig. 2B panel II). Infection efficiency of *ago1* single, double or triple mutants by TuMV-GFP was similar to that of wild type plants (Fig. 2A panels II and III), and infection efficiency of *ago1–27 ago2–1* double and *ago1–27 ago2–1 ago10–5* triple mutants by TuMV-AS9-GFP was similar to that of *dcl2–1 dcl3–1 dcl4–2* plants used as susceptible control (Fig. 2A panel II). Thus, both the lack of TuMV-AS9-GFP infection in single *ago1* mutants and the lack of systemic infection of inflorescence in *ago1–27 ago2–1* double mutants were not due to pleiotropic effects.

Surprisingly, systemic infection of inflorescence tissue was detected in the *ago1–27 ago10–5* double mutant and *ago1–27 ago2–1 ago10–5* triple mutant plants (Fig. 2B panel III and Table 3). Among all single and combination *ago* mutants tested, only those containing both *ago1* and *ago10* defects exhibited movement to, and accumulation in, inflorescences. However, while TuMV-AS9-GFP was detected in all inflorescence clusters of the *dcl2–1 dcl3–1 dcl4–2* triple mutant reference, in *ago1–27 ago10–5* and in *ago1–27 ago2–1 ago10–5* TuMV-AS9-GFP was detected only in 4% and 14% of the inflorescence clusters, respectively (Table 3). In inflorescences of *ago1–27 ago10–5* and *ago1–27 ago2–1 ago10–5* plants with visible GFP fluorescence, TuMV-AS9-GFP CP accumulated to 5% and 10% relative to the *dcl2–1 dcl3–1 dcl4–2* triple mutant (Fig. 2B panel III).

Collectively, the genetic analysis of local and systemic infection using TuMV-AS9-GFP revealed two sets of AGOs that limit infection. In inoculated rosette and noninoculated cauline leaves, AGO2 plays a major antiviral role, while AGO5, AGO7 and AGO10 play minor roles that are non-additive with AGO2. In noninoculated inflorescence tissues, AGO1 and AGO10 play overlapping or redundant antiviral roles, but these functions likely account for only a fraction of the RNA-mediated antiviral activity. It is possible that other factors, including AGO proteins not analyzed here, have a role in protecting inflorescence tissue from virus infection. The scope of subsequent AGO analyses was restricted to the functions of AGO1, AGO2 and AGO10 in the presence and absence of functional HC-Pro.

### Differential association of AGO2 with viral siRNAs in the presence and absence of functional HC-Pro

We hypothesized that AGO proteins with anti-TuMV activity associate with TuMV-derived siRNAs. This idea was tested first with epitope-tagged AGO2 in plants inoculated with parental TuMV or HC-Pro-defective TuMV-AS9 (lacking GFP) [23]. AGO2 immunoprecipitation and small RNA sequence analyses were done using transgenic *A*. *thaliana* expressing a triple-hemagglutinin (HA) epitope-tagged, catalytically inactive form of AGO2 (HA-AGO2_DAD_). The second of three aspartic acid residues of AGO2 was substituted with alanine; this substitution eliminates antiviral activity of AGO2, but preserves both the siRNA-binding and target RNA-binding functions [31]. These experiments require the use of plants lacking AGO2-mediated antiviral functions, as infection by TuMV-AS9 would otherwise be blocked (Figs. 1 and 2) [31].

Small RNAs from the input (pre-immunoprecipitated) and HA-AGO2_DAD_ co-immunoprecipitated fractions from inoculated rosette leaves and noninoculated inflorescences of TuMV-infected plants were analyzed from duplicate biological samples. Only reads that matched to either the *A*. *thaliana* or TuMV genomes without mismatches were analyzed (S1 Table). For each individual sample, read counts were scaled with respect to the total number of adaptor-parsed reads (reads per million) for the corresponding flow cell (eight individual samples). In mock-inoculated plants, a small number of reads from the input fractions mapped to TuMV (S1–S4 Tables, and S1 Fig). The source of these reads could be contamination, sequencing error, or portions of the *A*. *thaliana* genome. Based on the number of reads from mock-inoculated plants mapping to the TuMV genome, the false positive rate (proportion of parsed reads artifactually mapping to TuMV) was estimated to be between 9.8X10^-6^ and 1.0X10^-4^, which should not have affected subsequent analyses.

**Fig 3:**
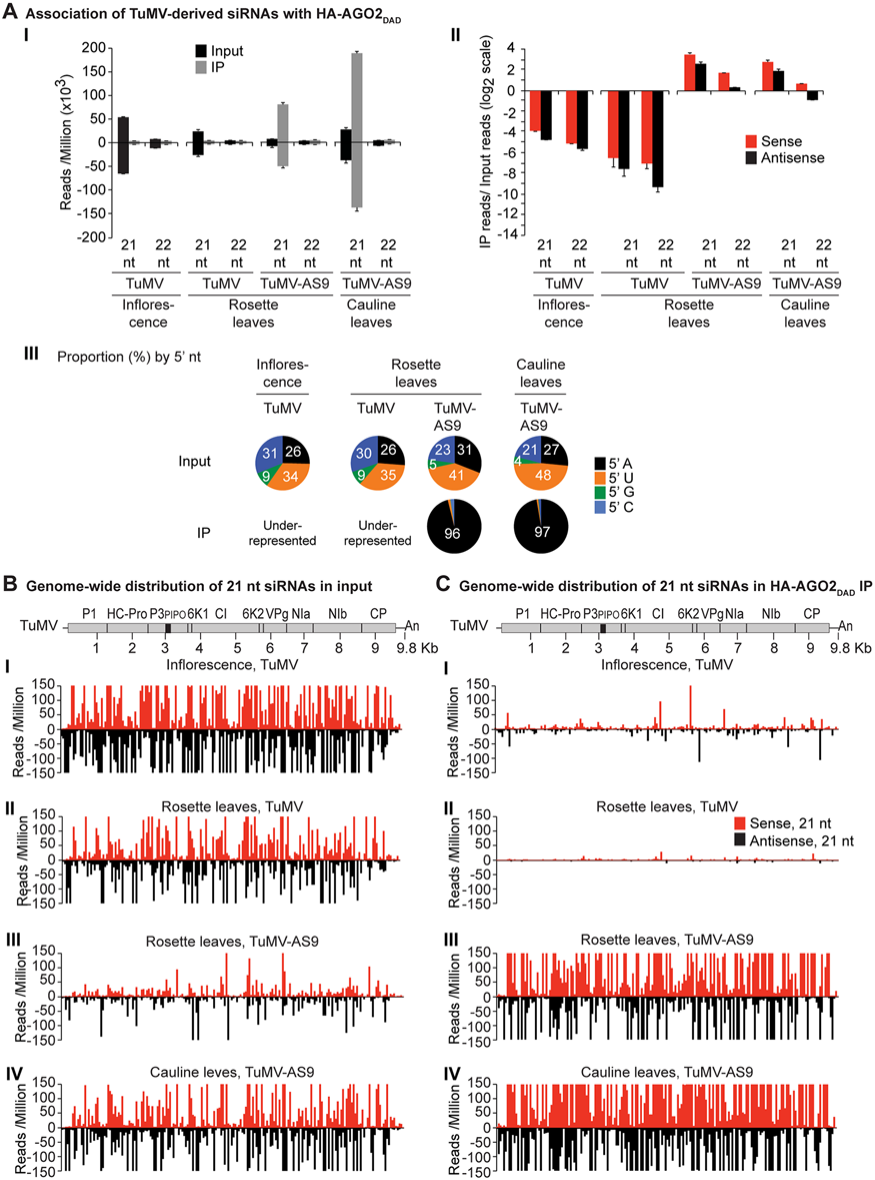
Profile of endogenous and TuMV-derived siRNAs in plants expressing HA-AGO2_DAD_ in an *ago2–1* background. Values are average and SE from two biological replicates normalized to reads per million. Inoculated rosette leaf and systemically infected cauline leaf samples were collected at 7 and 15 dpi, respectively. Inflorescence samples were collected at 10 dpi. (A) Panel I: number of reads by size, class, and polarity, for TuMV-derived siRNAs in input and HA-AGO2_DAD_ IP. Panel II: for 21 and 22 nt TuMV-derived siRNAs, enrichment in HA-AGO2_DAD_ IP. Enrichment is defined as immunoprecipitate (IP) reads/ input reads, expressed on a log_2_ scale. Panel III: proportion (in percentage) of 5’ nt in 21 nt and 22 nt TuMV-derived siRNAs by fraction. Numbers were rounded to the nearest integer. (B) and (C) TuMV genome-wide distribution of 21 nt TuMV-derived siRNAs in input (B) and HA-AGO2_DAD_ IP (C). Panel I: TuMV-infected inflorescence. Panel II: TuMV-inoculated rosette leaves. Panel III: rosette leaves inoculated with TuMV-AS9. Panel IV: cauline leaves systemically infected with TuMV-AS9. Reads were plotted for each 1 nt position. The scale was capped at 150 reads.

**Fig 4:**
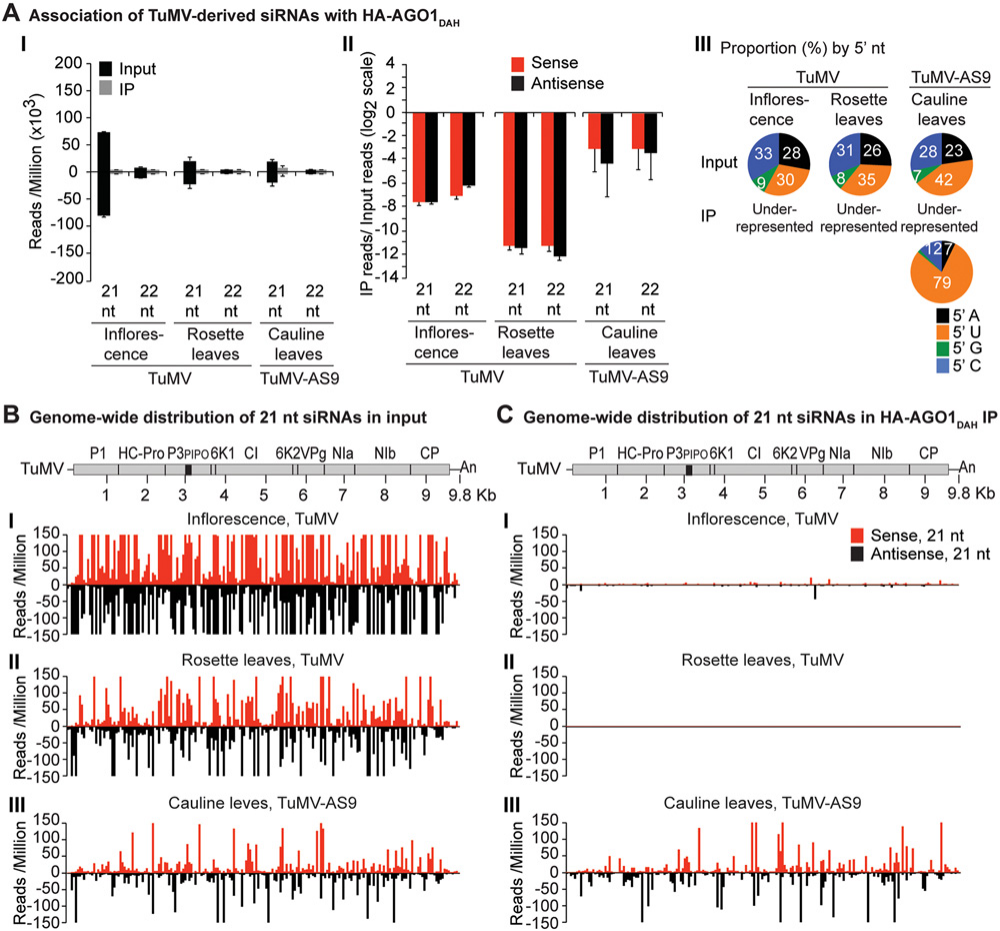
Profile of endogenous and TuMV-derived siRNAs in plants expressing HA-AGO1_DAH_ in an *ago2–1* background. Labels are as in Fig. 3. Inflorescence samples were collected at 10 dpi. Inoculated rosette leaf and systemically infected cauline leaf samples were collected at 7 and 15 dpi, respectively.

**Fig 5:**
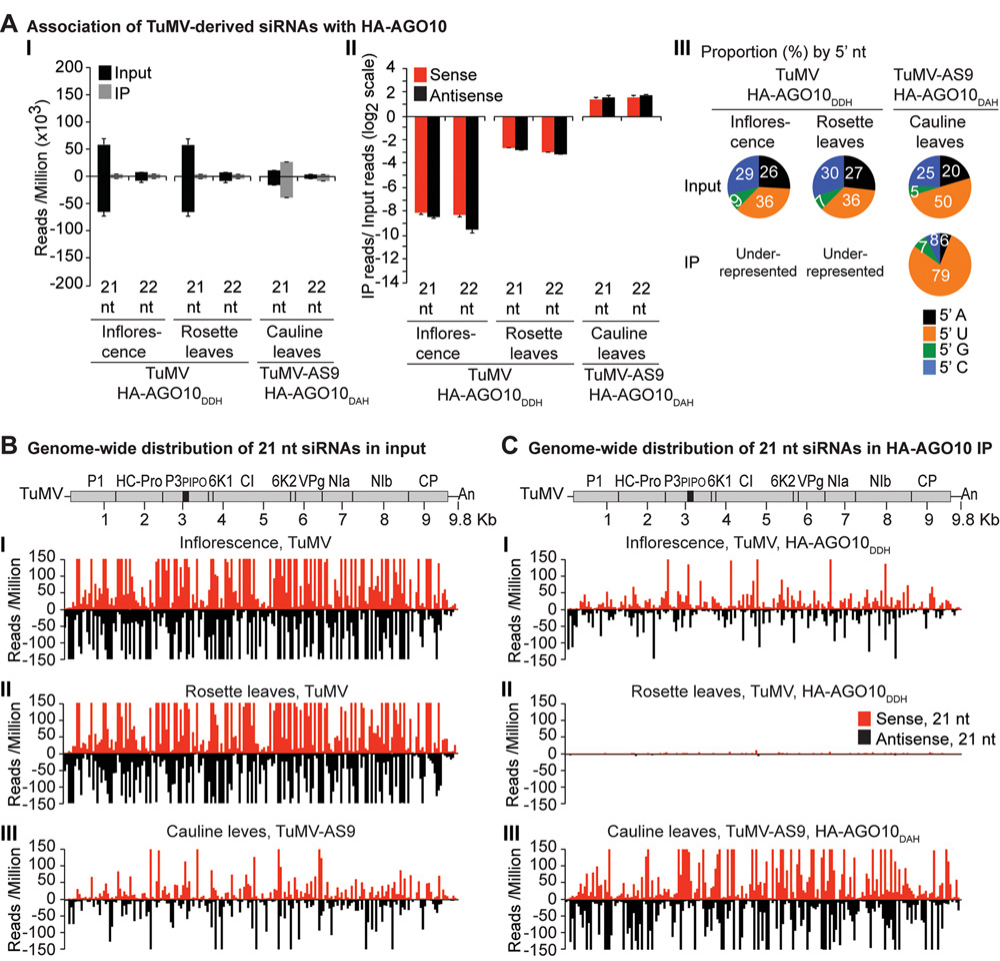
Profile of endogenous and TuMV-derived siRNAs in plants expressing HA-AGO10. Labels are as in Fig. 3. Catalytically active HA-AGO10_DDH_ and catalytic mutant HA-AGO10_DAH_ were expressed in a wild-type Col-0 (*AGO2*) or *ago2–1* background, respectively. Inflorescence samples were collected at 10 dpi. Inoculated rosette leaf and systemically infected cauline leaf samples were collected at 7 and 15 dpi, respectively.

**Fig 6:**
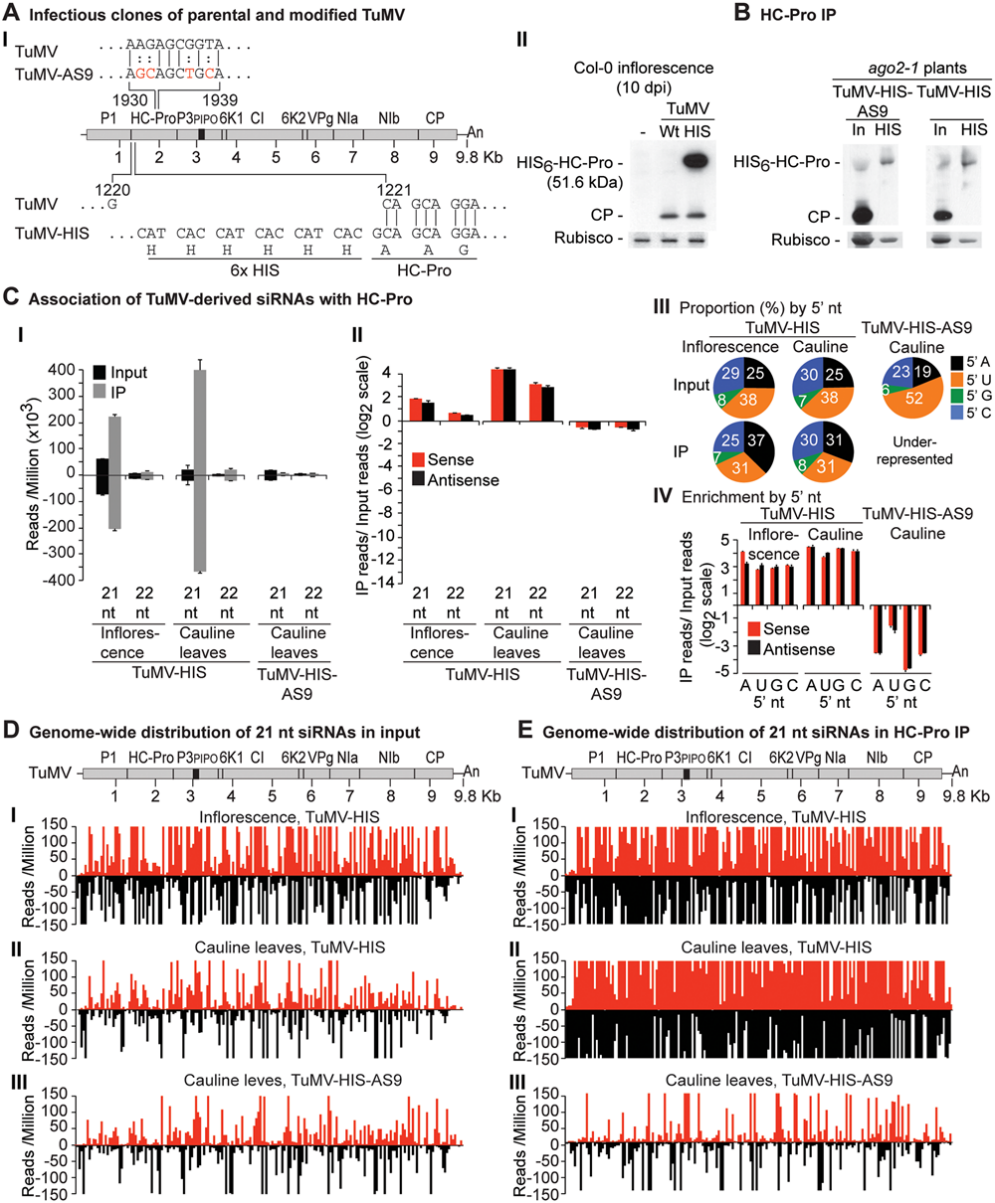
Profile of TuMV-derived siRNAs in plants infected with TuMV-HIS or TuMV-HIS-AS9. (A) Panel I: schematic representation of the TuMV genome and modified clones with an AS9 mutation and a 6xHIS tag (TuMV-HIS). Coordinates correspond to wild-type TuMV. The 6xHIS tag fused in frame to HC-Pro is underlined. Panel II: representative blot CP and HC-Pro accumulation in inflorescence of Col-0 at 10 dpi. (B) CP and HC-Pro accumulation in input and HC-Pro (wild-type and AS9) immunoprecipitation from cauline leaves of *ago2–1* plants. Samples from plants infected with TuMV-HIS or TuMV-HIS-AS9 were collected at 10 and 15 dpi, respectively. 6.25 μg of total protein or 10 μl of immunoprecipitate (IP) were loaded for TuMV-HIS input and IP samples, respectively. Amounts were doubled for TuMV-HIS-AS9 input and IP. (C) Panel I: number of reads by size, class, and polarity, for TuMV-derived siRNAs in input and wild-type or AS9 HC-Pro IP. Panel II: enrichment in HC-Pro IP as in Fig. 3. Panel III: proportion (in percentage) of 5’ nt in 21 nt and 22 nt TuMV-derived siRNAs by fraction. Panel IV: bars show the enrichment of TuMV-derived siRNAs by 5’ nt and polarity. (D) and (E) TuMV genome-wide distribution of 21 nt TuMV-derived siRNAs in input (D) and HC-Pro IP (E). Reads were plotted for each 1 nt position. The scale was capped at 150 reads.

In input fractions from TuMV-infected plants expressing HA-AGO2_DAD_, the proportion of reads mapping to the *A*. *thaliana* genome, as opposed to TuMV, varied from 77% (averaged across replicates) to 84% for different tissues (S1A Fig). Sequences mapping to TuMV were mainly 21-nt and 22-nt (S1A Fig). Accordingly, the detailed analyses for HA-AGO2_DAD_ and other proteins (discussed below) were focused on 21-nt (Figs 3–6) and 22-nt sequences (S3–S7) Figs.

Endogenous *A*. *thaliana* 21-nt small RNAs were enriched in HA-AGO2_DAD_ immunoprecipitates from leaves or inflorescence of mock-inoculated (4.5 to 10 fold) or TuMV-infected samples (2.7 to 6.3 fold) (S2A Fig). Enriched sequences in HA-AGO2_DAD_ immunoprecipitates had predominantly a 5’A nucleotide, as previously reported for AGO2-associated small RNAs [52, 53], or a 5’U nucleotide (S2A Fig). Specific miRNA, miRNA* and trans-acting siRNA (tasiRNA) populations were enriched in HA-AGO2_DAD_ immunoprecipitates from both mock-inoculated (2.3 to 31 fold), and to a lesser extent, TuMV-infected (1.8 to 16 fold) rosette leaves (S8A Fig). MicroRNA read counts for input and immunoprecipitates from this and subsequent analyses are provided in S1 Dataset. MiR390 and miR393* were shown previously to co-immunoprecipitate with AGO2 [35, 52]. In mock-inoculated and TuMV-infected rosette leaves, the number of miR390 reads in HA-AGO2_DAD_ immunoprecipitates was 260 and 65 fold higher, respectively, than in the corresponding input samples. Similarly, miR393* reads were enriched 125 and 60 fold in HA-AGO2_DAD_ immunoprecipitates from mock-inoculated and TuMV-infected rosette leaves, respectively. Therefore, enrichment of *A*. *thaliana* small RNA populations that are known to be associated with AGO2 occurred as expected.

In TuMV-inoculated rosette leaves, and systemically infected inflorescence, virus-derived siRNAs were abundant, representing 17% and 23%, respectively, of mapped reads in input samples (S1A Fig). Reads mapped to both sense (genomic strand) and antisense strands across the entire TuMV genome. However, both 21- and 22-nt TuMV-derived siRNAs were depleted in HA-AGO2_DAD_ immunoprecipitates (Fig. 3A panels I and II, Fig. 3B and 3C panels I and II, and S3 Fig); only a small number of individual TuMV-derived siRNAs were marginally enriched.

In leaves of TuMV-AS9-infected plants, endogenous *A*. *thaliana* small RNAs were again enriched (2.7 to 4.6 fold) in HA-AGO2_DAD_ immunoprecipitates, with patterns expected of AGO2-associated small RNAs (S2A Fig). Virus-derived siRNAs represented 7% or 16% of mapped reads in input samples from inoculated rosette leaves or systemically infected cauline leaves, respectively (S1A Fig). However, in striking contrast to TuMV-infected samples, both 21- and 22-nt TuMV-AS9-derived siRNAs were highly enriched relative to TuMV-derived siRNAs in HA-AGO2_DAD_ immunoprecipitates from both inoculated rosette leaves and systemically infected cauline leaves (Fig. 3A panels I and II, Fig. 3B and 3C panels III and IV, and S3 Fig). Among co-immunoprecipitated siRNAs, those containing a 5’A were overrepresented (Fig. 3A panel III). Association of AGO2 with siRNAs derived from TuMV-AS9, but not from TuMV, was verified by small RNA northern blot assays (S9 Fig). These results indicate that programming of AGO2 with TuMV-derived siRNAs is inhibited in the presence of active HC-Pro.

### Differential association of AGO1 and AGO10 with viral siRNAs in the presence and absence of functional HC-Pro

A similar experimental design was used to test the association of tagged AGO1 and AGO10 with TuMV and TuMV-AS9-derived siRNAs. To enable infection by suppressor-deficient TuMV-AS9, transgenic *A*. *thaliana* plants expressing catalytically defective HA-AGO1_DAH_ [31] or HA-AGO10_DAH_ were produced in the TuMV-AS9-permissive *ago2–1* background. Phenotypic defects associated to catalytic mutant HA-AGO1_DAH_ were more severe in an *ago1–25* mutant that in a wild-type (*AGO1*) background [31]. Effects of catalytically defective HA-AGO10_DAH_ on plant phenotype were not known, so transgenic *A*. *thaliana* plants expressing catalytically active HA-AGO10_DDH_ in a wild-type Col-0 background were also generated. Transgenic lines were inoculated with TuMV or TuMV-AS9 and samples from inoculated rosette leaves and systemically infected cauline leaves or inflorescences were collected from biological replicates. Small RNAs from input samples and immunoprecipitated fractions were sequenced, and reads were mapped and counts were scaled as described above. Tagged versions of AGO1 and AGO10 associated with small RNAs with a 5’U, as expected (S2B and S2C Fig panel II) [36, 52–54], and the proportion of *A*. *thaliana* and TuMV-derived siRNAs (S1B and S1C Fig) was similar to the observed in plants expressing HA-AGO2 (S1A Fig).

In mock-inoculated samples, endogenous *A*. *thaliana* 21-nt small RNAs were enriched 5 to 15 fold, and 5 to 7 fold, in HA-AGO1_DAH_ and HA-AGO10_DAH_ immunoprecipitates, respectively. In TuMV- and TuMV-AS9-infected samples, *A*. *thaliana* 21-nt small RNAs were enriched 5 and 15 fold, respectively, in HA-AGO1_DAH_ immunoprecipitates (S2B Fig panel I). In TuMV-infected samples, *A*. *thaliana* 21-nt small RNAs were enriched 1.5 and 2.5 fold in HA-AGO10_DDH_ immunoprecipitates from inflorescences and rosette leaves, respectively (S2C Fig panel I). In TuMV-AS9-infected samples, *A*. *thaliana* 21-nt small RNAs were enriched 7 fold in HA-AGO10_DAH_ immunoprecipitates from cauline leaves (S2C Fig panel I). Sequences with a 5’U were enriched with both AGOs (panel II in S2B and S2C Fig), as expected [36, 52–54]. MiRNAs were enriched in HA-AGO1_DAH_ and HA-AGO10_DAH_ immunoprecipitates from both mock-inoculated (7 to 50 fold) and TuMV-infected (3 to 25 fold) samples, while miRNA* and tasiRNA populations were variable (S8B and S8C Fig). For example, miR166 reads were enriched 30 and 45 fold in HA-AGO1_DAH_ immunoprecipitates from inflorescences of mock-inoculated and TuMV-infected plants, respectively. MiR168 reads were likewise enriched 20 and 12 fold. MiR166 reads were enriched 900 and 60 fold in HA-AGO10_DAH_ immunoprecipitates from mock-inoculated and TuMV-infected plants, respectively, in agreement with previous observations [36].

In rosette and inflorescence tissues from each of the transgenic lines, TuMV infection triggered abundant 21- and 22-nt siRNAs that originated from sense and antisense strands across the entire viral genome (Figs. 4B and 5B). However, as with HA-AGO2_DAD_ immunoprecipitates, TuMV-derived siRNAs were depleted in both HA-AGO1_DAH_ (Fig. 4A-4C panels I and II, and S4 Fig) and HA-AGO10_DDH_ (Fig. 5A-5C panels I and II, and S5 Fig) immunoprecipitates. By contrast, in plants infected with suppressor-deficient TuMV-AS9, virus-derived siRNAs were enriched in HA-AGO10_DAH_ immunoprecipitates (Fig. 5A panels I and II, Fig. 5B and 5C panels III, and S5 Fig), and had predominantly a 5’U nucleotide (Fig. 5A panel III). Individual highly enriched sequences were distributed across the TuMV-AS9 genome (Fig. 5C panel III and S5 Fig), suggesting that AGO10 may target all regions of TuMV-AS9 genome. TuMV-AS9-derived siRNAs were present in HA-AGO1_DAH_ immunoprecipitates at a higher level than in immunoprecipitates from plants infected with parental TuMV, although the overall population of TuMV-AS9-derived siRNAs was depleted relative to the input fraction (Fig. 4A panels I and II, Fig. 4B and 4C panel III, and S4 Fig). Only a few individual sequences were enriched; these sequences had predominantly a 5’U nucleotide (Fig. 4A panel III). Because depletion of TuMV-AS9-derived siRNAs in HA-AGO1_DAH_ immunoprecipitates was 60 to 1,200 fold lower than in TuMV-infected samples, we reasoned that AGO1 does interact with virus-derived siRNAs, but to a lesser extent than both AGO2 and AGO10.

### HC-Pro associates with siRNAs derived from the entire TuMV genome

Results described above show that AGO1, AGO2 and AGO10 associate at low levels with parental TuMV-derived siRNAs. In contrast, AGO2 and AGO10, and to a much lesser extent AGO1, associate with siRNAs derived from the suppressor-deficient TuMV-AS9 genome. Only two residues (R238A and V240A) in HC-Pro differ between TuMV and TuMV-AS9 (Fig. 6A panel I) [23, 38]. We hypothesized that i) HC-Pro associates with siRNAs-derived from the entire TuMV genome and sequesters them from AGO proteins, and ii) the AS9 mutation in HC-Pro reduces siRNA-binding activity. HC-Pro is known to have small RNA-binding activity [39, 43, 44, 55], but the extent to which it binds siRNAs in the context of TuMV infection has not been described. To measure the extent to which HC-Pro binds small RNA using the immunoprecipitation assay, we introduced an N-terminal 6xHistidine tag (HIS_6_) in the context of the TuMV (TuMV-HIS) and TuMV-AS9 (TuMV-HIS-AS9) genomes (Fig. 6A panel I). The addition of HIS_6_ to HC-Pro did not affect viral coat protein accumulation (Fig. 6A panel II), but enabled specific immunoprecipitation of HC-Pro from plants infected with TuMV-HIS and TuMV-HIS-AS9 (Fig. 6B).

Small RNAs from input and immunoprecipitated fractions obtained from plants inoculated with TuMV-HIS and TuMV-HIS-AS9 were sequenced. Because TuMV-HIS-AS9 accumulated more slowly than TuMV-HIS, TuMV-HIS samples were collected earlier than TuMV-HIS-AS9 samples (10 and 15 dpi, respectively), and twice as much input and immunoprecipitate materials for TuMV-HIS-AS9 samples were analyzed. The longer infection time and doubling of materials for TuMV-HIS-AS9 resulted in similar protein levels for HIS-HC-Pro and HIS-HC-Pro-AS9 input and immunoprecipitate fractions (Fig. 6B).

Endogenous *A*. *thaliana* small RNAs were depleted in suppressor-deficient HC-Pro-AS9 immunoprecipitates. Similarly, 22-, 23- and 24-nt *A*. *thaliana* endogenous small RNAs were depleted in wild-type HC-Pro immunoprecipitates (S6A Fig). In samples from systemically infected inflorescence or cauline leaves, *A*. *thaliana* endogenous 21-nt small RNAs were marginally enriched (2 fold) or depleted, respectively, in wild-type HC-Pro immunoprecipitates (S6A Fig). While miRNAs were depleted, miRNA* and tasiRNAs were enriched in HC-Pro immunoprecipitates (S6B–S6C Fig). Specifically, reads corresponding to miR390 and miR390* were enriched 8 and 64 fold, respectively, in wild-type HC-Pro immunoprecipitates. MiR166 reads were depleted 5 fold, whereas miR166* reads were enriched 16 fold in wild-type HC-Pro immunoprecipitates.

In contrast with results obtained for HA-AGO1_DAH_, HA-AGO2_DAD_ and HA-AGO10_DDH_ from TuMV-infected plants (compare panel I in Fig. 3C-5C to Fig. 6E), TuMV-derived siRNAs were highly enriched in HIS-HC-Pro immunoprecipitates from cauline leaves and inflorescence (Fig. 6C panels I and II, and Fig. 6D and 6E panels I and II). No 5’ nt preference was evident (Fig. 6C panels III and IV). HIS-HC-Pro associated preferentially with 21-nt over 22-nt siRNAs in samples from both cauline leaves and inflorescences (Fig. 6C, 6D-E panels I and II, and S7 Fig). In contrast, TuMV-HIS-AS9-derived siRNAs from across the genome were depleted in the HIS-HC-Pro-AS9 immunoprecipitates from systemically infected cauline leaves; only a few individual sequences were enriched (Fig. 6C panels I and II, 6D and 6E panel III, and S7 Fig). These results indicate that wild-type HC-Pro associates with TuMV-derived siRNAs, and that the AS9 mutation disrupts this association. We concluded that HC-Pro interferes with antiviral silencing, at least in part, by sequestering TuMV-derived siRNAs and preventing their association with antiviral AGO proteins. Suppression activity of HC-Pro is not tissue specific and affects AGO1, AGO2, AGO10 and possibly other AGO proteins.

## Discussion

Genetic and co-immunoprecipitation analyses were combined to reveal that i) several AGOs function as anti-TuMV defense modules in *A*. *thaliana*, ii) viral siRNAs generally fail to load into AGO proteins with antiviral functions during wild-type TuMV infection, and iii) HC-Pro sequesters viral siRNA away from AGOs with antiviral functions.

### Functions of AGO-small RNA complexes in anti-TuMV defense

**Fig 7:**
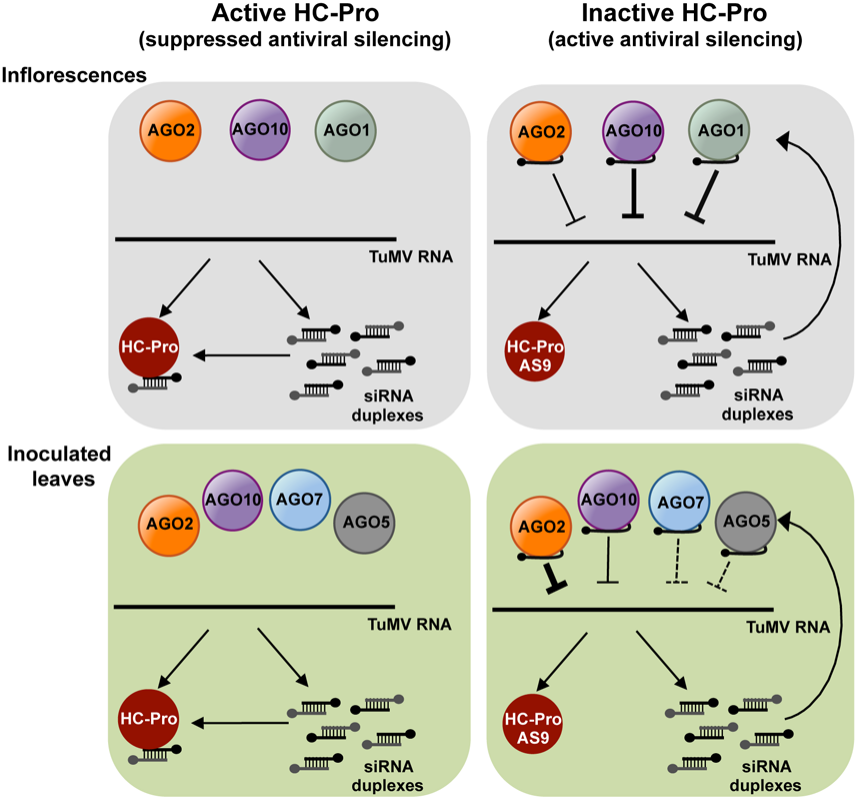
A model for direct action of *A*. *thaliana* AGO proteins in anti-TuMV defense. AGO-mediated antiviral silencing is suppressed through sequestration of TuMV-derived siRNAs by silencing suppressor HC-Pro (left panels), in both inoculated leaves and inflorescences. In the absence of active HC-Pro (right panels), AGO2, AGO10 and, to a lesser extent AGO1, associate with TuMV-AS9-derived siRNAs to potentially repress TuMV RNAs through slicing or translational repression. AGO2 protects leaves from TuMV infection and movement, with non-additive contributions by AGO10, AGO5 and AGO7. Redundant activities of AGO10 and AGO1 protect inflorescence from TuMV infection, with an additive contribution by AGO2.

AGO proteins target endogenous transcripts to regulate plant development and innate immunity [2, 56], which may indirectly affect susceptibility to viruses. It is likely, however, that at least some AGO proteins with an antiviral role are programmed with virus-derived siRNA to directly target viral RNA [8, 10, 57, 58]. The genetic analysis described here revealed several AGO proteins that participate in modular fashion during anti-TuMV defense (Fig. 7). AGO2 has the most influential role in protecting inoculated rosette and cauline leaves (Fig. 1), while AGO1 and AGO10 have genetically redundant roles in protecting inflorescence tissues. A larger proportion of *ago1 ago2 ago10* triple mutants than *ago1 ago10* double mutants were systemically infected (Table 3), perhaps suggesting that AGO2 also contributes to restricting virus spread to inflorescences.

The antiviral effects of different AGO proteins in different tissues may depend on a number of factors, including expression patterns, AGO-interacting partners, small RNA binding preferences, or subcellular localization. Microarray data suggest that *AGO10* and *AGO1* are expressed more strongly than *AGO2* in flowers and meristems [59]. However, *AGO1* and *AGO10* transcript levels are also higher than *AGO2* transcript levels in rosette leaves. Therefore, expression levels alone do not explain the effectiveness of individual AGOs in different organs. It is conceivable that modular, tissue-specific functionality is controlled by AGO-interacting or AGO-promoting factors that are tissue-specific. In *ago1 ago10* double mutants, systemic infection of inflorescences could be partially restricted because AGO2 limits virus accumulation in leaves, acts directly in inflorescences, or functions in both of these tissues.

Direct down-regulation of viral RNA requires that AGOs bind virus-derived siRNAs (or endogenous small RNAs complementary to a given viral genome) and then viral RNA, followed by slicing of the viral RNA, repression of translation, and/or recruitment of factors for silencing amplification. Results described here show that AGO2, AGO10 and at much lower levels AGO1 associate with TuMV-AS9-derived siRNA in the absence of HC-Pro (Fig. 3C panels III and IV, and Figs. 4C and 5C panel III). AGO2-mediated slicing of viral RNAs could be a significant anti-viral mechanism, as catalytically defective forms of AGO2 lack anti-TuMV activity [31]. Evidence of direct targeting of TuMV RNA by AGO1 and AGO10 is lacking. In other studies, AGO1 was reported to bind small RNAs derived from *Turnip yellow mosaic virus* and CMV strains Fny and NT9 [20], but not CMV strain I17F or *Crucifer-infecting tobamovirus* [60]. The basis for differential interaction of TuMV-derived siRNAs and AGO1, AGO2 and AGO10 is not clear. It is possible that different AGOs have privileged access to viral siRNAs. In this context, AGO1 pools may have limited access to viral siRNAs during TuMV infection.

In inoculated rosette leaves of *ago2* mutant and *dcl2 dcl3 dcl4* triple mutant plants, TuMV-AS9 accumulated to comparable levels (Figs. 1 and 2). In contrast, accumulation of TuMV-AS9 was consistently lower in cauline leaves and inflorescences of all *ago* mutants tested, including the *ago1 ago2 ago10* triple mutant, compared to the respective tissues in *dcl2 dcl3 dcl4* mutant plants. If it is assumed that all small RNA-mediated antiviral activity is lost in the *dcl* triple mutant, then it is reasonable to conclude that all antiviral silencing in inoculated rosette leaves is mediated by AGO2. The far greater effect of the *dcl* mutations, relative to the *ago* mutations, in systemic tissues, especially inflorescences, argues that the combined effects of AGO1, AGO2 and AGO10 account for only a small proportion of overall anti-TuMV silencing activity. This could indicate that other AGO proteins that were not tested here, or that were not tested in the right genetic combinations, play specific roles in systemic tissues. It could also mean that DCL proteins play a more dominant, direct antiviral role in systemic tissues, as suggested by genetic analyses with CMV [4, 29], BMV [30], PVX [27], *Tobacco rattle virus* [61], TCV [3, 6, 33, 62], *Cauliflower mosaic virus*, *Cabbage leaf curl virus*, and *Oil rape mosaic virus* [63].

Different antiviral AGO proteins may also have distinct effects on amplification of secondary, virus-derived siRNAs, which may be important for production of systemic signals [2, 7, 13, 64]. Full anti-TuMV silencing requires both RDR1 and RDR6 [23], presumably for production of dsRNA from viral RNA. If this occurs like dsRNA formation during tasiRNA biogenesis, then RDR proteins may be recruited to viral RNA after targeting by AGO-small RNA complexes [52, 65–68]. Given the role of AGO1-small RNA complexes in triggering formation of several families of tasiRNA, AGO1 could conceivably play a trigger role for secondary viral siRNA.

The interpretation of *ago1* mutant susceptibility experiments is challenging because of the pleiotropic developmental phenotypes of *ago1* hypomorphic mutants and the large number of genes that are dysregulated when AGO1 is disrupted. In particular, disruption of AGO1-miR403 activity increases *AGO2* mRNA and protein levels [26, 69], which could result in a net increase in virus resistance, even if AGO1 directly targets viral RNA.

Other AGOs might also have indirect roles in anti-TuMV defense, perhaps by affecting expression of defense-related genes [35, 56, 70]. Expression of potyviral HC-Pro [45], infection with TCV [26], and infection with *Pseudomonas syringae* [35] result in increased *AGO2* expression; *AGO*2 regulates expression of *MEMB12* [35] and possibly other genes. AGO2 also associates with virus-activated endogenous siRNAs [56]. The significance of AGO2-dependent gene regulation for virus infection, if any, is not yet clear.

### Suppression of antiviral silencing by HC-Pro

Multiple virus-encoded suppressors of RNA silencing target AGO1 [16, 17, 20, 21, 33, 60], and P25 from PVX interact with AGO2, AGO3 and AGO4 [17] although the biological significance of this interaction remains to be elucidated. During TuMV infection, no evidence was obtained to indicate that AGO1, AGO2 or AGO10 were destabilized or otherwise down-regulated. Each AGO accumulated to normal levels.

TuMV-infected plants accumulate large amounts of virus-derived siRNAs that map across the entire genome (Figs. 3B, 4B, 5B, 6B, and S3–S5 Figs) [23], and co-immunoprecipitation and high-throughput sequencing showed that HC-Pro associates with viral siRNAs in leaf and inflorescence tissue (Fig. 6E panels I and II). Viral siRNAs associate with HC-Pro without a 5’ nt preference (Fig. 6C panels III and IV). Importantly, HC-Pro was shown to sequester viral siRNAs away from AGO1, AGO2 and AGO10 (Figs. 3C, 4C and 5C panels I and II), leading to the obvious proposal that HC-Pro interferes with antiviral silencing by preventing AGOs from loading with virus-derived siRNAs (Fig. 7). Mutant HC-Pro-AS9 is deficient in associating with viral siRNAs (Fig. 6C-E panels III, and S6 Fig), and concomitantly loses silencing suppression activity.

The basis for sequestration of siRNAs by HC-Pro is not yet clear. HC-Pro may outcompete AGOs for siRNAs. Alternatively, HC-Pro may intercept viral siRNAs prior to AGO loading, perhaps due to subcellular localization properties. Further analyses will be necessary to resolve this issue.

## Materials and Methods

### DNA plasmids

Recombinant plasmids were made as follows.

#### *pCB-TuMV-HIS* and *pCB-TuMV-HIS-AS9*

To introduce a 6xHIS (HIS_6_) tag on HC-Pro, two PCR fragments were amplified from pCB-TUMV [16] using two sets of primers: TuMV764 d(AGGACGGTGCACAGAATATGC) and E101-B2Rev d(CCAGAAGTTGGCTCCTGCTGCGTGATGGTGATGGTGATGACCTGCCTGGTGATAGACACAGCTAGCACTAAAGTGCAC); and E101-B2For d(GTGCACTTTAGTGCTAGCTGTGTCTATCACCAGGCAGGTCATCACCATCACCATCACGCAGCAGGAGCCAACTTCTGG) and TuMV-GFP-2873 d(CGCCTGATTCTGTTGTGACAC). The two PCR fragments were stitched into a final PCR product using primers TuMV764 and TuMV-GFP-2873. The final PCR product was digested with StuI-AgeI and used to replace the StuI-AgeI fragment in pCB-TuMV, creating pCB-TuMV-HIS. The same insert was used to replace the StuI-AgeI fragment in pCB-TuMV-AS9 [16], to generate pCB-TuMV-HIS-AS9. Both HIS_6_-tagged clones have a NIa cleavage site between P1 and the HIS_6_-tag on HC-Pro.

#### *pMDC99-pAGO10*:*3xHA-AGO10*_*DDH*_ *and pMDC99-pAGO10*:*3xHA-AGO10*_*DAH*_

For in-frame N-terminal 3xHA-tagging of wild-type *AGO10*_*DDH*_ in its natural genomic context, a 9072 bp genomic region was TOPO cloned into pENTR (Invitrogen) in two pieces: an upstream region (with primers caccGATTTCTATAAAAAATAcattcc and CTCGAGGCGGCCGCCCATGGTTTTTGTTGTTTGGATTTTC) and the coding and downstream regions (with HA-containing forward primer caccATGGCCTATCCTTATGATGTACCTGATTATGCCTACCCATACGACGTTCCAGACTACGCTTACCCATACGACGTTCCAGACTACGCTCCGATTAGGCAAATGAAAGATAG and reverse primer cctagaattgacgggtttagatcg). The first piece was ligated upstream of the second using a *NotI* site in pENTR and a *NcoI* site created by the cloning primers, producing pENTR-pAGO10–3xHA-AGO10_DDH_. To disrupt the AGO10 PIWI domain catalytic triad, A2384 in the coding sequence of pENTR-pAGO10:3xHA-AGO10_DDH_ was mutated to G by GENEWIZ Inc., causing amino acid substitution D795A to generate pENTR-pAGO10–3xHA-AGO10_DAH_. Transgenes from pENTR-pAGO10–3xHA-AGO10_DDH_ and pENTR-pAGO10–3xHA-AGO10_DAH_ were LR recombined into binary vector pMDC99 [71], producing pMDC99-pAGO10:3xHA-AGO10_DDH_ and pMDC99-pAGO10:3xHA-AGO10_DAH_, respectively.

### Plant materials

All *Arabidopsis thaliana* plants used in this study (including mutant lines and transgenic lines) descended from the Columbia-0 (Col-0) accession, and were grown under long day (16 h light/8 h dark) at 22°C. The following single mutant lines were described before: *ago1–25* and *ago1–27* [25], *ago2–1* [72], *ago3–2* [32], *ago4–2* [73], *ago5–2* [32], *ago6–3* [32], *zip-1* [74], *ago8–1* [32], and *ago9–5* (SALK_126176). T-DNA insertion mutant GABI_818H06 (*ago10–5*) was obtained from The GABI KAT project [75]. Homozygous mutants were confirmed by PCR-based genotyping using a three-primer reaction: one on the left border, one in the flanking DNA, and one in the T-DNA insertion site [76]. Lack of *AGO10* expression in homozygous plants was confirmed by RT-PCR using oligos AGO10_qF (GGTATTCAGGGAACAAGCAG) and AGO10_qR (GCTGGAGGAACTATAGAGACCG). Double and triple *ago* mutants were generated by crossing. *dcl2–1 dcl3–1 dcl4–2* triple mutants have been described [3].

Transgenic *A*. *thaliana* plants expressing HA-tagged AGO1 or AGO2 catalytic mutants from their native promoters have been described [31]. Transgenic *A*. *thaliana* plants expressing HA-tagged wild-type or catalytic mutant AGO10 from its native promoter were made by dipping Col-0 plants in *Agrobacterium tumefaciens* GV3101 carrying the pMDC99-pAGO10:3xHA-AGO10_DDH_ or pMDC99-pAGO10:3xHA-AGO10_DAH_ constructs as described [77]. Transgenic plants were grown on MS medium containing hygromycin (50 mg/ml) for 7 days, transferred to soil, and maintained in greenhouse conditions. Catalytic mutant HA-AGO1_DAH_, HA-AGO2_DAD_ and HA-AGO10_DAH_ and wild-type HA-AGO10_DDH_ transgenes were introduced into *ago2–1* by crossing.

### Virus infection assays

*A*. *thaliana* plants were inoculated with TuMV-GFP, TuMV-AS9-GFP, wild-type TuMV, TuMV-AS9, TuMV-HIS, or TuMV-HIS-AS9 as described previously [23]. Local and systemic infection by TuMV-GFP or TuMV-AS9-GFP was determined by GFP fluorescence under UV illumination. To measure coat protein (CP) or HIS_6_-tagged HC-Pro (HIS-HC-Pro) accumulation, at 15 days post inoculation (dpi), four noninoculated cauline leaves or five inflorescence clusters per plant were randomly collected and pooled into a single sample. Four biological replicates were randomly collected per virus-plant genotype combination. Samples were ground in glycine buffer [78] at a ratio of 0.5 mL per 1g of leaf, or 0.25 mL per five inflorescence clusters. Protein extracts were normalized to 0.5 mg/mL. For western blot assays, 6.25 μg or 1.5 μg of total protein were used for leaf or inflorescence samples, respectively. Immunoblotting and chemiluminescence detection were done as described [23]. TuMV CP was detected using antibody PVAS-134 (1:40,000) and HIS-HC-Pro was detected using anti-HIS antibody 27E8-HRP (Cell Signaling) at a 1:5,000 dilution. Ponceau staining of the large subunit of rubisco was used as a loading control. Unless otherwise indicated, CP and HIS-HC-Pro were detected simultaneously on the same blot. In experiments involving HA-tagged AGOs, HA-AGO, CP and HIS-HC-Pro were detected on the same blot. The top part of the blot, containing proteins larger than 70 kDA was incubated with anti-HA antibodies, to detect HA-AGOs. The part of the blot containing proteins between 70 and 27 kDa was probed for CP and HIS-HC-Pro.

### Immunoprecipitation of HA-tagged ARGONAUTES and HIS-tagged HC-Pro

Immunoprecipitation of epitope tagged proteins was performed as described [31] with minor adjustments. Briefly, one gram of leaf or inflorescence tissue was ground in 6 ml of lysis buffer. Lysates were pre-cleared by incubating with protein A agarose (Roche) beads (0.8 mL per 1g of tissue) for 30 min at 4°C, and beads were not treated with P1 nuclease. For immunoblot detection of proteins (CP, HA-AGOs or HIS-HC-Pro), 6.25 μg or 1.5 μg of total protein from leaf or inflorescence samples were used, respective. From the immunoprecipitated beads 5% of the samples was diluted with 38 μl of 2x protein dissociation buffer, and 5 to 15 μL used for immunoblotting. For small RNA northern blotting, 15 μg were used from the input fractions and 25% of the RNA immunoprecipitate fraction (HA or HIS).

### Small RNA library construction for high-throughput sequencing

Small RNA libraries from mock-inoculated or TuMV-infected plants, input or immunoprecipitate (HA or HIS) fractions were generated using sequencing-by synthesis technology (Illumina High Seq 2000) as described [31, 79]. For input fractions, 50 μg of total RNA were fractionated by electrophoresis. The area from 16 to 26 nt was sliced and used for small RNA purification. 30 ng of small RNAs were used to make the libraries from total fraction. 50% of the immunoprecipitated RNA was used without fractionation to make libraries from immunoprecipitate fractions. For each treatment, small RNA libraries were made independently from two biological replicates. Bar-coded PCR amplification primers were used for multiplexing purposes. Eight individual samples were multiplexed and run in a single flow cell.

### Bioinformatic analysis of small RNA libraries

Bioinformatic analysis of endogenous and TuMV-derived siRNAs was as described [23, 31, 80]. After removing 5’ and 3’ adaptors, sequences were aligned to the *A*. *thaliana* genome and to the TuMV genome. Only sequences with a perfect match were used for downstream analysis. For each sample, reads were normalized per 1,000,000 total reads (RPM), including all size classes. Enrichment with respect to the immunoprecipitate was calculated as the ratio of reads in the immunoprecipitate to reads in the input, and expressed on a log_2_ scale.

### Accession numbers

Sequence data from this article can be found in Gene Expression Omnibus (GEO, http://www.ncbi.nlm.nih.gov/geo) accession number GSE64911.

## Supporting Information

S1 Fig Proportion of *A*. *thaliana* endogenous and TuMV-derived small RNAs in mock-inoculated and in TuMV-infected plants. Samples for immunoprecipitation were collected from inflorescence 10 (dpi), rosette leaves (7 dpi), or cauline leaves (15 dpi). Numbers are the relative abundance, in percentage, of reads mapping to *A*. *thaliana* or to TuMV with respect to the total number of reads with a perfect match to either genome. Proportion of TuMV-derived siRNAs by size class is indicated by numbers (percentage) in color pie charts. Numbers were rounded to the nearest integer. Plants expressing (A) HA-AGO2_DAD_, (B) HA-AGO1_DAH_ from an *ago2–1* background and were inoculated with wild-type TuMV or TuMV-AS9. (C) HA-AGO10_DDH_ or HA-AGO10_DAD_ were expressed from a AGO2 or *ago2–1* background, respectively. (D) Wild-type Col-0 or single *ago2–1* mutant plants were inoculated with TuMV-HIS or TuMV-HIS-AS9. Color codes are as in (A).
(TIF)

S2 Fig Association of endogenous siRNAs with HA-tagged AGO1, AGO2 and AGO10. Values are average and SE from two biological replicates normalized to reads per million. Inoculated rosette leaf, systemically infected cauline leaves or inflorescence samples were collected at 7, 15 or 10 dpi, respectively. (A) HA-AGO2_DAD_ in an *ago2–1* background. Panel I: enrichment [immunoprecipitate (IP) reads/ input reads, expressed in a log_2_ scale] of endogenous (21 to 24 nt) small RNAs in mock-inoculated plants and in plants infected with wild-type TuMV or TuMV-AS9. In the scale was capped at 4 and at-4. Panel II: proportion (in percentage) of 5’ nt in 21 nt and 22 nt small RNAs in input and in HA-AGO2_DAD_ immunoprecipitated (IP) fractions. Numbers were rounded to the nearest integer. (B) HA-AGO1_DAH_ in an *ago2–1* background. Labels for panels I and II are as in (A). (C) Catalytically active HA-AGO10_DDH_ and catalytic mutant HA-AGO10_DAH_ were expressed in a wild-type Col-0 (*AGO2*) or *ago2–1* background, respectively. Labels for panels I and II are as in (A).
(TIF)

S3 Fig TuMV genome-wide distribution and enrichment of 22-nt TuMV-derived siRNAs in plants expressing HA-AGO2_DAD_ in an *ago2–1* background. Values are average and SE from two biological replicates normalized to reads per million. Scale was capped at 150. Inoculated rosette leaf and systemically infected cauline leaf samples were collected at 7 and 15 dpi, respectively. Inflorescence samples were collected at 10 dpi. (A) and (B) TuMV genome-wide distribution of 22 nt TuMV-derived siRNAs in input (A) and in HA-AGO2_DAD_ immunoprecipitated (IP) fractions (B). Scale was capped at 150.
(TIF)

S4 Fig TuMV genome-wide distribution and enrichment of 22-nt TuMV-derived siRNAs in plants expressing HA-AGO1_DAH_ in an *ago2–1* background. Values are average and SE from two replicates normalized to reads per million. Inflorescence samples were collected at 10 dpi. Inoculated rosette leaf and systemically infected cauline leaf samples were collected at 7 and 15 dpi, respectively. (A) and (B) TuMV genome-wide distribution of 22 nt TuMV-derived siRNAs in input (A) and in HA-AGO1_DAH_ immunoprecipitated fractions (IP) (B). Scale was capped at 150.
(TIF)

S5 Fig TuMV genome-wide distribution and enrichment of 22-nt TuMV-derived siRNAs in plants expressing HA-AGO10_DDH_ or HA-AGO10_DAH_. Values are average and SE from two replicates normalized to reads per million. Inflorescence samples were collected at 10 dpi. Inoculated rosette leaf and systemically infected cauline leaf samples were collected at 7 and 15 dpi, respectively. (A) and (B) TuMV genome-wide distribution of 22 nt TuMV-derived siRNAs in input (A) and in HA-AGO10 immunoprecipitated (IP) fractions (B). Scale was capped at 150.
(TIF)

S6 Fig Association of endogenous siRNAs (21–24-nt) with HC-Pro in plants infected with TuMV-HIS or TuMV-HIS-AS9. Values are average and SE from two biological replicates normalized to reads per million. Inflorescence and cauline leaf samples from plants infected with TuMV-HIS were collected at 10 dpi. Cauline leaf samples from plants infected with TuMV-HIS-AS9 were collected at 15 dpi. (A) Number of reads of endogenous *A*. *thaliana* siRNAs by size class in input and HC-Pro immunoprecipitated (IP) fractions from inflorescence and cauline leaves. (B) Number of reads for miRNAs, miRNA* and tasiRNAs in input and mock or HC-Pro IP. (C) Enrichment (IP reads/ Input reads, expressed in a log_2_ scale) of miRNAs, miRNA* and tasiRNAs (*TAS*) in mock or HC-Pro IP. Scales was capped at 3 and -3.
(TIF)

S7 Fig TuMV genome-wide distribution and enrichment of 22-nt TuMV-derived siRNAs in Col-0 or *ago2–1* plants infected with TuMV-HIS or TuMV-HIS-AS9. Values are average and SE from two biological replicates normalized to reads per million. Scale was capped at 500. Inflorescence samples were from Col-0 plants at 10 dpi. Cauline leaf samples were from single *ago2–1* mutant plants infected with TuMV-HIS or TuMV-HIS-AS9 at 10 or 15 dpi, respectively. (A) and (B) TuMV genome-wide distribution of 22 nt TuMV-derived siRNAs in input (A) or immunoprecipitated (IP) fractions of wild-type or AS9 HC-Pro.
(TIF)

S8 Fig Association of *A*. *thaliana* miRNAs, miRNA* and tasiRNAs with HA-tagged AGO2_DAD_, AGO1_DAH_, AGO10_DDH_ or AGO10_DAH_. Transgenic HA-AGO1_DAH_ and HA-AGO2 _DAD_ were expressed from an *ago2–1* background. Transgenic HA-AGO10_DDH_ and HA-AGO10_DAH_ were expressed from a wild-type Col-0 (*AGO2*) or an *ago2–1* background, respectively. Plants were mock-inoculated or infected with TuMV or with TuMV-AS9. Rosette leaf and samples were collected at 7 dpi. Cauline leaf and inflorescence samples were collected at 15 and 10 dpi, respectively. Values are average and SE from two biological replicates. The histograms show average fold enrichment in AGO IP (IP reads/ input reads, expressed in log_2_ scale) of miRNAs, miRNA* and tasiRNAs. A) HA-AGO2_DAD_ IP. B) HA-AGO1_DAH_ IP, and C) HA-AGO10_DDH_ or HA-AGO10_DAH_ IP.
(TIF)

S9 Fig Association of HA-AGO1_DAH_ and HA-AGO2_DAD_ with endogenous and virus-derived siRNAs. Blots show accumulation of CP, HA-AGO, and virus-derived small RNAs in immunoprecipitation (IP) fractions of HA-AGO1_DAH_ and HA-AGO2_DAD_ from cauline leaves (1g) at 15 dpi. HA-AGO1_DAH_ and HA-AGO2_DAD_ were expressed from transgenic *ago2–1* plants. Mock-inoculated plants and non-trangenic single *ago2–1* mutants were used as controls. Representative blots showing accumulation of HA-AGOs, CP, TuMV-derived siRNAs (CI) and selected miRNAs in input and HA-AGO immunoprecipitation fractions (IPs). TuMV CP and HA-AGO were detected by immunoblotting in input and IP fractions. TuMV-derived siRNAs were detected with a DIG-labeled probe made by random priming of cDNA corresponding to CI. miR390 and miR168 were used as IP controls, and U6 as loading control. Endogenous siRNAs were detected with DIG-labeled oligonucleotides. Duplicated blots were stripped and re-probed. A) IP of HA-AGO1_DAH_ and HA-AGO2_DAD_ from cauline leaves of plants infected with wt TuMV. Panel I: protein accumulation in input samples. Panel II: protein accumulation in IP fractions. B) IP of HA-AGO1_DAH_ and HA-AGO2_DAD_ from cauline leaves of plants infected with suppressor-deficient TuMV-AS9. Panels I and II are as in (A).
(TIF)

S1 Table Abundance of endogenous *A*. *thaliana* and TuMV-derived small RNAs of all size classes in input and HA-AGO2 immunoprecipitation fractions. (DOCX)

S2 Table Abundance of endogenous *A*. *thaliana* and TuMV-derived small RNAs of all size classes in input and AGO1 immunoprecipitation fractions. (DOCX)

S3 Table Abundance of endogenous *A*. *thaliana* and TuMV-derived small RNAs of all size classes in input and AGO10 immunoprecipitation fractions. (DOCX)

S4 Table Abundance of endogenous *A*. *thaliana* and TuMV-derived small RNAs of all size classes in input and HC-Pro immunoprecipitation fractions. (DOCX)

S1 Dataset MicroRNA read counts for input and immunoprecipitates of HA-AGO2, AGO1, AGO10 and HC-Pro. (XLSX)

## References

[1] Ding SW, Voinnet O. Antiviral immunity directed by small RNAs. Cell. 2007;130(3):413–26. doi: 10.1016/j.cell.2007.07.039 17693253 10.1016/j.cell.2007.07.039 PMC2703654

[2] Pumplin N, Voinnet O. RNA silencing suppression by plant pathogens: defence, counter- defence and counter-counter-defence. Nat Rev Microbiol. 2013;11(11):745–60. doi: 10.1038/nrmicro3120. 24129510 10.1038/nrmicro3120

[3] Deleris A, Gallego-Bartolome J, Bao J, Kasschau KD, Carrington JC, Voinnet O.Hierarchical action and inhibition of plant Dicer-like proteins in antiviral defense. Science. 2006;313(5783):68–71. doi: 10.1126/science.1128214. 16741077 10.1126/science.1128214

[4] Diaz-Pendon JA, Li F, Li WX, Ding SW. Suppression of antiviral silencing by cucumber mosaic virus 2b protein in Arabidopsis is associated with drastically reduced accumulation of three classes of viral small interfering RNAs. Plant Cell. 2007;19(6):2053–63. doi: 10.1105/tpc.106.047449 17586651 10.1105/tpc.106.047449 PMC1955711

[5] Curtin SJ, Watson JM, Smith NA, Eamens AL, Blanchard CL, Waterhouse PM. The roles of plant dsRNA-binding proteins in RNAi-like pathways. FEBS Lett. 2008;582(18):2753–60. doi: 10.1016/j.febslet.2008.07.004 18625233 10.1016/j.febslet.2008.07.004

[6] Qu F, Ye X, Morris TJ. Arabidopsis DRB4, AGO1, AGO7, and RDR6 participate in a DCL4-initiated antiviral RNA silencing pathway negatively regulated by DCL1. Proc Natl Acad Sci U S A. 2008;105(38):14732–7. doi: 10.1073/pnas.0805760105 18799732 10.1073/pnas.0805760105 PMC2567185

[7] Bologna NG, Voinnet O. The diversity, biogenesis, and activities of endogenous silencing small RNAs in Arabidopsis. Annu Rev Plant Biol. 2014;65:473–503. 10.1146/annurev- arplant-050213–035728 24579988 10.1146/annurev-arplant-050213-035728

[8] Schuck J, Gursinsky T, Pantaleo V, Burgyan J, Behrens SE. AGO/RISC-mediated antiviral RNA silencing in a plant in vitro system. Nucleic Acids Res. 2013;41(9):5090–103. 10.1093/nar/gkt193 23535144 10.1093/nar/gkt193 PMC3643602

[9] Brodersen P, Sakvarelidze-Achard L, Bruun-Rasmussen M, Dunoyer P, Yamamoto YY, Sieburth L, et al Widespread translational inhibition by plant miRNAs and siRNAs. Science. 2008;320(5880):1185–90. 10.1126/science.1159151 18483398 10.1126/science.1159151

[10] Ciomperlik JJ, Omarov RT, Scholthof HB. An antiviral RISC isolated from Tobacco rattle virus-infected plants. Virology. 2011;412(1):117–24. 10.1016/j.virol.2010.12.018 21272908 10.1016/j.virol.2010.12.018 PMC3056891

[11] Iwakawa HO, Tomari Y. Molecular Insights into microRNA-Mediated Translational Repression in Plants. Mol Cell. 2013;52(4):591–601. Epub 2013/11/26. 10.1016/j.molcel.2013.10.033 24267452 10.1016/j.molcel.2013.10.033

[12] Huntzinger E, Izaurralde E. Gene silencing by microRNAs: contributions of translational repression and mRNA decay. Nature reviews Genetics. 2011;12(2):99–110. 10.1038/nrg2936 21245828 10.1038/nrg2936

[13] Szittya G, Burgyan J. RNA interference-mediated intrinsic antiviral immunity in plants. Current topics in microbiology and immunology. 2013;371:153–81. 10.1007/978–3- 642–37765–5_6 23686235 10.1007/978-3-642-37765-5_6

[14] Incarbone M, Dunoyer P. RNA silencing and its suppression: novel insights from in planta analyses. Trends Plant Sci. 2013;18(7):382–92. 10.1016/j.tplants.2013.04.001 23684690 10.1016/j.tplants.2013.04.001

[15] Baumberger N, Tsai CH, Lie M, Havecker E, Baulcombe DC. The Polerovirus silencing suppressor P0 targets ARGONAUTE proteins for degradation. Curr Biol. 2007;17(18):1609–14. 10.1016/j.cub.2007.08.039 17869110 10.1016/j.cub.2007.08.039

[16] Bortolamiol D, Pazhouhandeh M, Marrocco K, Genschik P, Ziegler-Graff V. The Polerovirus F box protein P0 targets ARGONAUTE1 to suppress RNA silencing. Curr Biol. 2007;17(18):1615–21. 10.1016/j.cub.2007.07.061 17869109 10.1016/j.cub.2007.07.061

[17] Chiu MH, Chen IH, Baulcombe DC, Tsai CH. The silencing suppressor P25 of Potato virus X interacts with Argonaute1 and mediates its degradation through the proteasome pathway. Mol Plant Pathol. 2010;11(5):641–9. 10.1111/j.1364–3703.2010.00634.x 20696002 10.1111/j.1364-3703.2010.00634.x PMC6640501

[18] Csorba T, Lozsa R, Hutvagner G, Burgyan J. Polerovirus protein P0 prevents the assembly of small RNA-containing RISC complexes and leads to degradation of ARGONAUTE1. Plant J. 2010;62(3):463–72. 10.1111/j.1365–313X.2010.04163.x 20128884 10.1111/j.1365-313X.2010.04163.x

[19] Derrien B, Baumberger N, Schepetilnikov M, Viotti C, De Cillia J, Ziegler-Graff V, et al Degradation of the antiviral component ARGONAUTE1 by the autophagy pathway. Proc Natl Acad Sci U S A. 2012;109(39):15942–6. 10.1073/pnas.1209487109 23019378 10.1073/pnas.1209487109 PMC3465452

[20] Zhang X, Yuan YR, Pei Y, Lin SS, Tuschl T, Patel DJ, et al Cucumber mosaic virus- encoded 2b suppressor inhibits Arabidopsis Argonaute1 cleavage activity to counter plant defense. Genes Dev. 2006;20(23):3255–68. 10.1101/gad.1495506 17158744 10.1101/gad.1495506 PMC1686603

[21] Giner A, Lakatos L, Garcia-Chapa M, Lopez-Moya JJ, Burgyan J. Viral protein inhibits RISC activity by argonaute binding through conserved WG/GW motifs. PLoS Pathog. 2010;6(7):e1000996 10.1371/journal.ppat.1000996 20657820 10.1371/journal.ppat.1000996 PMC2904775

[22] Nakahara KS, Masuta C. Interaction between viral RNA silencing suppressors and host factors in plant immunity. Curr Opin Plant Biol. 2014;20:88–95. 10.1016/j.pbi.2014.05.004 24875766 10.1016/j.pbi.2014.05.004

[23] Garcia-Ruiz H, Takeda A, Chapman EJ, Sullivan CM, Fahlgren N, Brempelis KJ, et al Arabidopsis RNA-dependent RNA polymerases and dicer-like proteins in antiviral defense and small interfering RNA biogenesis during Turnip Mosaic Virus infection. Plant Cell. 2010;22(2):481–96. 10.1105/tpc.109.073056 20190077 10.1105/tpc.109.073056 PMC2845422

[24] Vaucheret H. Plant ARGONAUTES. Trends Plant Sci. 2008;13(7):350–8. Epub 2008/05/30. S1360–1385(08)00138–6[pii] 10.1016/j.tplants.2008.04.007 18508405 10.1016/j.tplants.2008.04.007

[25] Morel JB, Godon C, Mourrain P, Beclin C, Boutet S, Feuerbach F, et al Fertile hypomorphic ARGONAUTE (ago1) mutants impaired in post-transcriptional gene silencing and virus resistance. Plant Cell. 2002;14(3):629–39. 11910010 10.1105/tpc.010358 PMC150585

[26] Harvey JJ, Lewsey MG, Patel K, Westwood J, Heimstadt S, Carr JP, et al An antiviral defense role of AGO2 in plants. PLoS One. 2011;6(1):e14639 10.1371/journal.pone.0014639 21305057 10.1371/journal.pone.0014639 PMC3031535

[27] Jaubert MJ, Bhattacharjee S, Mello AF, Perry KL, Moffett P. AGO2 mediates RNA silencing anti-viral defenses against Potato virus X in Arabidopsis. Plant physiology. 2011 Epub 2011/05/18. 10.1104/pp.111.178012 10.1104/pp.111.178012 PMC3135937 21576511

[28] Zhang X, Singh J, Li D, Qu F. Temperature-dependent survival of Turnip crinkle virus- infected arabidopsis plants relies on an RNA silencing-based defense that requires dcl2, AGO2, and HEN1. Journal of virology. 2012;86(12):6847–54. Epub 2012/04/13. 10.1128/JVI.00497–12 22496240 10.1128/JVI.00497-12 PMC3393596

[29] Wang XB, Jovel J, Udomporn P, Wang Y, Wu Q, Li WX, et al The 21-Nucleotide, but Not 22-Nucleotide, Viral Secondary Small Interfering RNAs Direct Potent Antiviral Defense by Two Cooperative Argonautes in Arabidopsis thaliana. The Plant cell. 2011;23(4):1625–38. Epub 2011/04/07. 10.1105/tpc.110.082305 21467580 10.1105/tpc.110.082305 PMC3101545

[30] Dzianott A, Sztuba-Solinska J, Bujarski JJ. Mutations in the antiviral RNAi defense pathway modify Brome mosaic virus RNA recombinant profiles. Molecular plant-microbe interactions: MPMI. 2012;25(1):97–106. Epub 2011/09/23. 10.1094/MPMI-05–11- 0137 21936664 10.1094/MPMI-05-11-0137

[31] Carbonell A, Fahlgren N, Garcia-Ruiz H, Gilbert KB, Montgomery TA, Nguyen T, et al Functional analysis of three Arabidopsis ARGONAUTES using slicer-defective mutants. The Plant cell. 2012;24(9):3613–29. Epub 2012/10/02. 10.1105/tpc.112.099945 10.1105/tpc.112.099945 PMC3480291 23023169

[32] Takeda A, Iwasaki S, Watanabe T, Utsumi M, Watanabe Y. The mechanism selecting the guide strand from small RNA duplexes is different among argonaute proteins. Plant Cell Physiol. 2008;49(4):493–500. 10.1093/pcp/pcn043 18344228 10.1093/pcp/pcn043

[33] Azevedo J, Garcia D, Pontier D, Ohnesorge S, Yu A, Garcia S, et al Argonaute quenching and global changes in Dicer homeostasis caused by a pathogen-encoded GW repeat protein. Genes Dev. 2010;24(9):904–15. 10.1101/gad.1908710 20439431 10.1101/gad.1908710 PMC2861190

[34] Wei W, Ba Z, Gao M, Wu Y, Ma Y, Amiard S, et al A role for small RNAs in DNA double- strand break repair. Cell. 2012;149(1):101–12. Epub 2012/03/27. 10.1016/j.cell.2012.03.002 22445173 10.1016/j.cell.2012.03.002

[35] Zhang X, Zhao H, Gao S, Wang WC, Katiyar-Agarwal S, Huang HD, et al Arabidopsis Argonaute 2 regulates innate immunity via miRNA393 (*)-mediated silencing of a Golgi- localized SNARE gene, MEMB12. Mol Cell. 2011;42(3):356–66. 10.1016/j.molcel.2011.04.010 21549312 10.1016/j.molcel.2011.04.010 PMC3101262

[36] Zhu H, Hu F, Wang R, Zhou X, Sze SH, Liou LW, et al Arabidopsis Argonaute10 specifically sequesters miR166/165 to regulate shoot apical meristem development. Cell. 2011;145(2):242–56. 10.1016/j.cell.2011.03.024 21496644 10.1016/j.cell.2011.03.024 PMC4124879

[37] Mallory AC, Hinze A, Tucker MR, Bouche N, Gasciolli V, Elmayan T, et al Redundant and specific roles of the ARGONAUTE proteins AGO1 and ZLL in development and small RNA-directed gene silencing. PLoS Genet. 2009;5(9):e1000646 10.1371/journal.pgen.1000646 19763164 10.1371/journal.pgen.1000646 PMC2730571

[38] Kasschau KD, Cronin S, Carrington JC. Genome amplification and long-distance movement functions associated with the central domain of tobacco etch potyvirus helper component-proteinase. Virology. 1997;228(2):251–62. 10.1006/viro.1996.8368 9123832 10.1006/viro.1996.8368

[39] Lakatos L, Csorba T, Pantaleo V, Chapman EJ, Carrington JC, Liu YP, et al Small RNA binding is a common strategy to suppress RNA silencing by several viral suppressors. EMBO J. 2006;25(12):2768–80. 10.1038/sj.emboj.7601164 10.1038/sj.emboj.7601164 PMC1500863 16724105

[40] Mallory AC, Reinhart BJ, Bartel D, Vance VB, Bowman LH. A viral suppressor of RNA silencing differentially regulates the accumulation of short interfering RNAs and micro- RNAs in tobacco. Proc Natl Acad Sci U S A. 2002;99(23):15228–33. 10.1073/pnas.232434999 12403829 10.1073/pnas.232434999 PMC137572

[41] Chapman EJ, Prokhnevsky AI, Gopinath K, Dolja VV, Carrington JC. Viral RNA silencing suppressors inhibit the microRNA pathway at an intermediate step. Genes Dev. 2004;18(10):1179–86. 10.1101/gad.1201204 15131083 10.1101/gad.1201204 PMC415642

[42] Kasschau KD, Xie Z, Allen E, Llave C, Chapman EJ, Krizan KA, et al P1/HC-Pro, a viral suppressor of RNA silencing, interferes with Arabidopsis development and miRNA unction. Dev Cell. 2003;4(2):205–17. 12586064 10.1016/s1534-5807(03)00025-x

[43] Schott G, Mari-Ordonez A, Himber C, Alioua A, Voinnet O, Dunoyer P. Differential effects of viral silencing suppressors on siRNA and miRNA loading support the existence of two distinct cellular pools of ARGONAUTE1. The EMBO journal. 2012;31(11):2553–65. Epub 2012/04/26. 10.1038/emboj.2012.92 22531783 10.1038/emboj.2012.92 PMC3365429

[44] Shiboleth YM, Haronsky E, Leibman D, Arazi T, Wassenegger M, Whitham SA, et al Theconserved FRNK box in HC-Pro, a plant viral suppressor of gene silencing, is required for small RNA binding and mediates symptom development. J Virol. 2007;81(23):13135–48. 10.1128/JVI.01031–07 17898058 10.1128/JVI.01031-07 PMC2169133

[45] Endres MW, Gregory BD, Gao Z, Foreman AW, Mlotshwa S, Ge X, et al Two plant viral suppressors of silencing require the ethylene-inducible host transcription factor RAV2 to block RNA silencing. PLoS Pathog. 2010;6(1):e1000729 10.1371/journal.ppat.1000729 20084269 10.1371/journal.ppat.1000729 PMC2800190

[46] Ala-Poikela M, Goytia E, Haikonen T, Rajamaki ML, Valkonen JP. Helper component proteinase of the genus Potyvirus is an interaction partner of translation initiation factors eIF(iso)4E and eIF4E and contains a 4E binding motif. J Virol. 2011;85(13):6784–94. 10.1128/JVI.00485–11 21525344 10.1128/JVI.00485-11 PMC3126533

[47] Anandalakshmi R, Marathe R, Ge X, Herr JM Jr., Mau C, Mallory A, et al A calmodulin- related protein that suppresses posttranscriptional gene silencing in plants. Science. 2000;290(5489):142–4. Epub 2000/10/06. 11021800 10.1126/science.290.5489.142

[48] Iki T, Yoshikawa M, Nishikiori M, Jaudal MC, Matsumoto-Yokoyama E, Mitsuhara I, et al In vitro assembly of plant RNA-induced silencing complexes facilitated by molecular chaperone HSP90. Mol Cell. 2010;39(2):282–91. 10.1016/j.molcel.2010.05.014 20605502 10.1016/j.molcel.2010.05.014

[49] Ballut L, Drucker M, Pugniere M, Cambon F, Blanc S, Roquet F, et al HcPro, a multifunctional protein encoded by a plant RNA virus, targets the 20S proteasome and affects its enzymic activities. J Gen Virol. 2005;86(Pt 9):2595–603. 10.1099/vir.0.81107–0 16099919 10.1099/vir.0.81107-0

[50] Soitamo AJ, Jada B, Lehto K. HC-Pro silencing suppressor significantly alters the gene expression profile in tobacco leaves and flowers. BMC Plant Biol. 2011;11:68 10.1186/1471–2229–11–68 21507209 10.1186/1471-2229-11-68 PMC3111369

[51] Lellis AD, Kasschau KD, Whitham SA, Carrington JC. Loss-of-susceptibility mutants of Arabidopsis thaliana reveal an essential role for eIF(iso)4E during potyvirus infection. Curr Biol. 2002;12(12):1046–51. Epub 2002/07/19. 10.1016/s0960-9822(02)00898-9 12123581

[52] Montgomery TA, Howell MD, Cuperus JT, Li D, Hansen JE, Alexander AL, et al Specificity of ARGONAUTE7-miR390 interaction and dual functionality in TAS3 trans- acting siRNA formation. Cell. 2008;133(1):128–41. Epub 2008/03/18. 10.1016/j.cell.2008.02.033 18342362 10.1016/j.cell.2008.02.033

[53] Mi S, Cai T, Hu Y, Chen Y, Hodges E, Ni F, et al Sorting of small RNAs into Arabidopsis argonaute complexes is directed by the 5' terminal nucleotide. Cell. 2008;133(1):116–27. 10.1016/j.cell.2008.02.034 18342361 10.1016/j.cell.2008.02.034 PMC2981139

[54] Wang H, Zhang X, Liu J, Kiba T, Woo J, Ojo T, et al Deep sequencing of small RNAs specifically associated with Arabidopsis AGO1 and AGO4 uncovers new AGO functions. Plant J. 2011;67(2):292–304. Epub 2011/04/05. 10.1111/j.1365–313X.2011.04594.x 10.1111/j.1365-313X.2011.04594.x PMC3135789 21457371

[55] Merai Z, Kerenyi Z, Kertesz S, Magna M, Lakatos L, Silhavy D. Double-stranded RNA binding may be a general plant RNA viral strategy to suppress RNA silencing. J Virol. 2006;80(12):5747–56. 10.1128/JVI.01963–05 16731914 10.1128/JVI.01963-05 PMC1472586

[56] Cao M, Du P, Wang X, Yu YQ, Qiu YH, Li W, et al Virus infection triggers widespread silencing of host genes by a distinct class of endogenous siRNAs in Arabidopsis. Proc Natl Acad Sci U S A. 2014;111(40):14613–8. 10.1073/pnas.1407131111 25201959 10.1073/pnas.1407131111 PMC4209997

[57] Omarov RT, Ciomperlik JJ, Scholthof HB. RNAi-associated ssRNA-specific ribonucleases in Tombusvirus P19 mutant-infected plants and evidence for a discrete siRNA-containing effector complex. Proc Natl Acad Sci U S A. 2007;104(5):1714–9. 10.1073/pnas.0608117104 17244709 10.1073/pnas.0608117104 PMC1785274

[58] Pantaleo V, Szittya G, Burgyan J. Molecular bases of viral RNA targeting by viral small interfering RNA-programmed RISC. J Virol. 2007;81(8):3797–806. 10.1128/JVI.02383–06 17267504 10.1128/JVI.02383-06 PMC1866121

[59] Schmid M, Davison TS, Henz SR, Pape UJ, Demar M, Vingron M, et al A gene expression map of Arabidopsis thaliana development. Nat Genet. 2005;37(5):501–6. 10.1038/ng1543 15806101 10.1038/ng1543

[60] Baumberger N, Baulcombe DC. Arabidopsis ARGONAUTE1 is an RNA Slicer that selectively recruits microRNAs and short interfering RNAs. Proc Natl Acad Sci U S A. 2005;102(33):11928–33. 10.1073/pnas.0505461102 16081530 10.1073/pnas.0505461102 PMC1182554

[61] Donaire L, Barajas D, Martinez-Garcia B, Martinez-Priego L, Pagan I, Llave C. Structural and genetic requirements for the biogenesis of tobacco rattle virus-derived small interfering RNAs. J Virol. 2008;82(11):5167–77. 10.1128/JVI.00272–08 18353962 10.1128/JVI.00272-08 PMC2395200

[62] Cao M, Ye X, Willie K, Lin J, Zhang X, Redinbaugh MG, et al The capsid protein of Turnip crinkle virus overcomes two separate defense barriers to facilitate systemic movement of the virus in Arabidopsis. J Virol. 2010;84(15):7793–802. 10.1128/JVI.02643–09 20504923 10.1128/JVI.02643-09 PMC2897622

[63] Blevins T, Rajeswaran R, Shivaprasad PV, Beknazariants D, Si-Ammour A, Park HS, et al Four plant Dicers mediate viral small RNA biogenesis and DNA virus induced silencing. Nucleic Acids Res. 2006;34(21):6233–46. 10.1093/nar/gkl886 17090584 10.1093/nar/gkl886 PMC1669714

[64] Wang N, Zhang D, Wang Z, Xun H, Ma J, Wang H, et al Mutation of the RDR1 gene caused genome-wide changes in gene expression, regional variation in small RNA clusters and localized alteration in DNA methylation in rice. BMC Plant Biol. 2014;14:177 10.1186/1471–2229–14–177 24980094 10.1186/1471-2229-14-177 PMC4083042

[65] Montgomery TA, Yoo SJ, Fahlgren N, Gilbert SD, Howell MD, Sullivan CM, et al AGO1- miR173 complex initiates phased siRNA formation in plants. Proc Natl Acad Sci U S A. 2008;105(51):20055–62. Epub 2008/12/11. 0810241105[pii]10.1073/pnas.0810241105 19066226 10.1073/pnas.0810241105 PMC2598728

[66] Cuperus JT, Carbonell A, Fahlgren N, Garcia-Ruiz H, Burke RT, Takeda A, et al Unique functionality of 22-nt miRNAs in triggering RDR6-dependent siRNA biogenesis from target transcripts in Arabidopsis. Nature structural & molecular biology. 2010;17(8):997–1003. Epub 2010/06/22. 10.1038/nsmb.1866 10.1038/nsmb.1866 PMC2916640 20562854

[67] Rajeswaran R, Aregger M, Zvereva AS, Borah BK, Gubaeva EG, Pooggin MM. Sequencing of RDR6-dependent double-stranded RNAs reveals novel features of plant siRNA biogenesis. Nucleic Acids Res. 2012;40(13):6241–54. 10.1093/nar/gks242 22434877 10.1093/nar/gks242 PMC3401431

[68] Chen HM, Chen LT, Patel K, Li YH, Baulcombe DC, Wu SH. 22-Nucleotide RNAs trigger secondary siRNA biogenesis in plants. Proc Natl Acad Sci U S A. 2010;107(34):15269–74. 10.1073/pnas.1001738107 20643946 10.1073/pnas.1001738107 PMC2930544

[69] Allen E, Xie Z, Gustafson AM, Carrington JC. microRNA-directed phasing during trans- acting siRNA biogenesis in plants. Cell. 2005;121(2):207–21. 10.1016/j.cell.2005.04.004 15851028 10.1016/j.cell.2005.04.004

[70] Bhattacharjee S, Zamora A, Azhar MT, Sacco MA, Lambert LH, Moffett P. Virus resistance induced by NB-LRR proteins involves Argonaute4-dependent translational control. The Plant journal: for cell and molecular biology. 2009;58(6):940–51. Epub 2009/02/18. 10.1111/j.1365–313X.2009.03832.x 19220787 10.1111/j.1365-313X.2009.03832.x

[71] Curtis MD, Grossniklaus U. A gateway cloning vector set for high-throughput functional analysis of genes in planta. Plant Physiol. 2003;133(2):462–9. 10.1104/pp.103.027979 14555774 10.1104/pp.103.027979 PMC523872

[72] Lobbes D, Rallapalli G, Schmidt DD, Martin C, Clarke J. SERRATE: a new player on the plant microRNA scene. EMBO Rep. 2006;7(10):1052–8. 10.1038/sj.embor.7400806 16977334 10.1038/sj.embor.7400806 PMC1618363

[73] Agorio A, Vera P. ARGONAUTE4 is required for resistance to Pseudomonas syringae in Arabidopsis. Plant Cell. 2007;19(11):3778–90. 10.1105/tpc.107.054494 17993621 10.1105/tpc.107.054494 PMC2174867

[74] Hunter C, Sun H, Poethig RS. The *Arabidopsis* heterochronic gene *ZIPPY* is an ARGONAUTE family member. Curr Biol. 2003;13(19):1734–9. 14521841 10.1016/j.cub.2003.09.004

[75] Kleinboelting N, Huep G, Kloetgen A, Viehoever P, Weisshaar B. GABI-Kat SimpleSearch: new features of the Arabidopsis thaliana T-DNA mutant database. Nucleic Acids Res. 2012;40(Database issue):D1211–5. 10.1093/nar/gkr1047 22080561 10.1093/nar/gkr1047 PMC3245140

[76] Alonso JM, Stepanova AN, Leisse TJ, Kim CJ, Chen H, Shinn P, et al Genome-wide insertional mutagenesis of *Arabidopsis thaliana* . Science. 2003;301(5633):653–7. 10.1126/science.1086391 12893945 10.1126/science.1086391

[77] Clough SJ, Bent AF. Floral dip: a simplified method for *Agrobacterium*-mediated transformation of *Arabidopsis thaliana* . Plant J. 1998;16(6):735–43. 10069079 10.1046/j.1365-313x.1998.00343.x

[78] Varallyay E, Valoczi A, Agyi A, Burgyan J, Havelda Z. Plant virus-mediated induction of miR168 is associated with repression of ARGONAUTE1 accumulation. EMBO J. 2010;29(20):3507–19. 10.1038/emboj.2010.215 20823831 10.1038/emboj.2010.215 PMC2964164

[79] Gilbert K, Faber N, Kasschau K, Chapman EJ, Carrington JC, Carbonell A. Preparation of Multiplexed Small RNA Libraries From Plants. Bioprotocol. 2014;4(21). 10.21769/bioprotoc.1275 PMC4675356 26661568

[80] Fahlgren N, Sullivan CM, Kasschau KD, Chapman EJ, Cumbie JS, Montgomery TA, et al Computational and analytical framework for small RNA profiling by high-throughput sequencing. RNA. 2009;15(5):992–1002. 10.1261/rna.1473809 19307293 10.1261/rna.1473809 PMC2673065

