# Supplementary figures and images for: Roles and Programming of Arabidopsis ARGONAUTE Proteins during Turnip Mosaic Virus Infection
Source: PLoS Pathog. 2015 Mar 25;11(3):e1004755. doi: 10.1371/journal.ppat.1004755 (PMC4373807; doi:10.1371/journal.ppat.1004755)

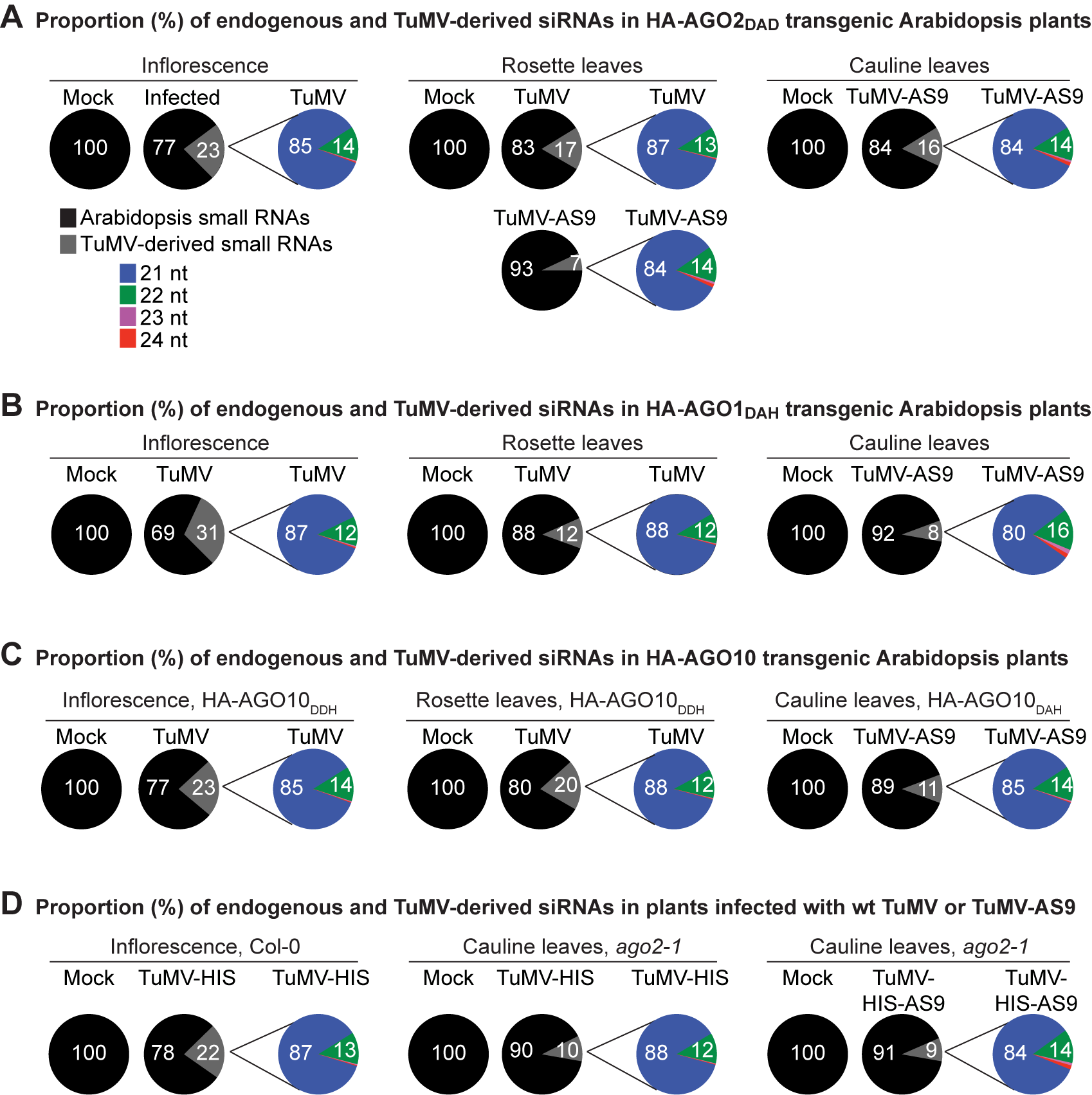

Supplement: S1 Fig — Samples for immunoprecipitation were collected from inflorescence 10 (dpi), rosette leaves (7 dpi), or cauline leaves (15 dpi). Numbers are the relative abundance, in percentage, of reads mapping to A. thaliana or to TuMV with respect to the total number of reads with a perfect match to either genome. Proportion of TuMV-derived siRNAs by size class is indicated by numbers (percentage) in color pie charts. Numbers were rounded to the nearest integer. Plants expressing (A) HA-AGO2DAD, (B) HA-AGO1DAH from an ago2–1 background and were inoculated with wild-type TuMV or TuMV-AS9. (C) HA-AGO10DDH or HA-AGO10DAD were expressed from a AGO2 or ago2–1 background, respectively. (D) Wild-type Col-0 or single ago2–1 mutant plants were inoculated with TuMV-HIS or TuMV-HIS-AS9. Color codes are as in (A). (TIF) [file ppat.1004755.s001.tif]

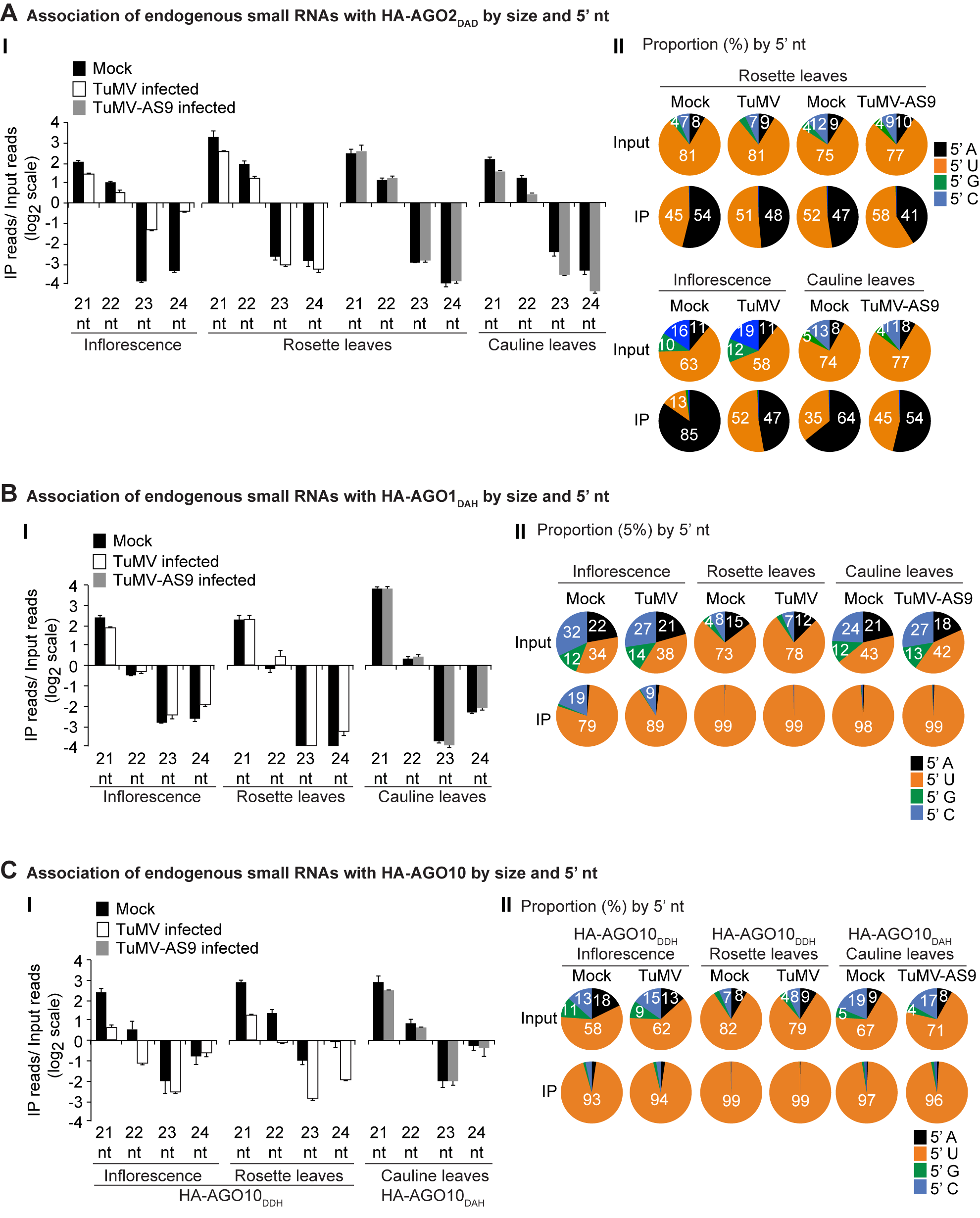

Supplement: S2 Fig — Values are average and SE from two biological replicates normalized to reads per million. Inoculated rosette leaf, systemically infected cauline leaves or inflorescence samples were collected at 7, 15 or 10 dpi, respectively. (A) HA-AGO2DAD in an ago2–1 background. Panel I: enrichment [immunoprecipitate (IP) reads/ input reads, expressed in a log2 scale] of endogenous (21 to 24 nt) small RNAs in mock-inoculated plants and in plants infected with wild-type TuMV or TuMV-AS9. In the scale was capped at 4 and at-4. Panel II: proportion (in percentage) of 5’ nt in 21 nt and 22 nt small RNAs in input and in HA-AGO2DAD immunoprecipitated (IP) fractions. Numbers were rounded to the nearest integer. (B) HA-AGO1DAH in an ago2–1 background. Labels for panels I and II are as in (A). (C) Catalytically active HA-AGO10DDH and catalytic mutant HA-AGO10DAH were expressed in a wild-type Col-0 (AGO2) or ago2–1 background, respectively. Labels for panels I and II are as in (A). (TIF) [file ppat.1004755.s002.tif]

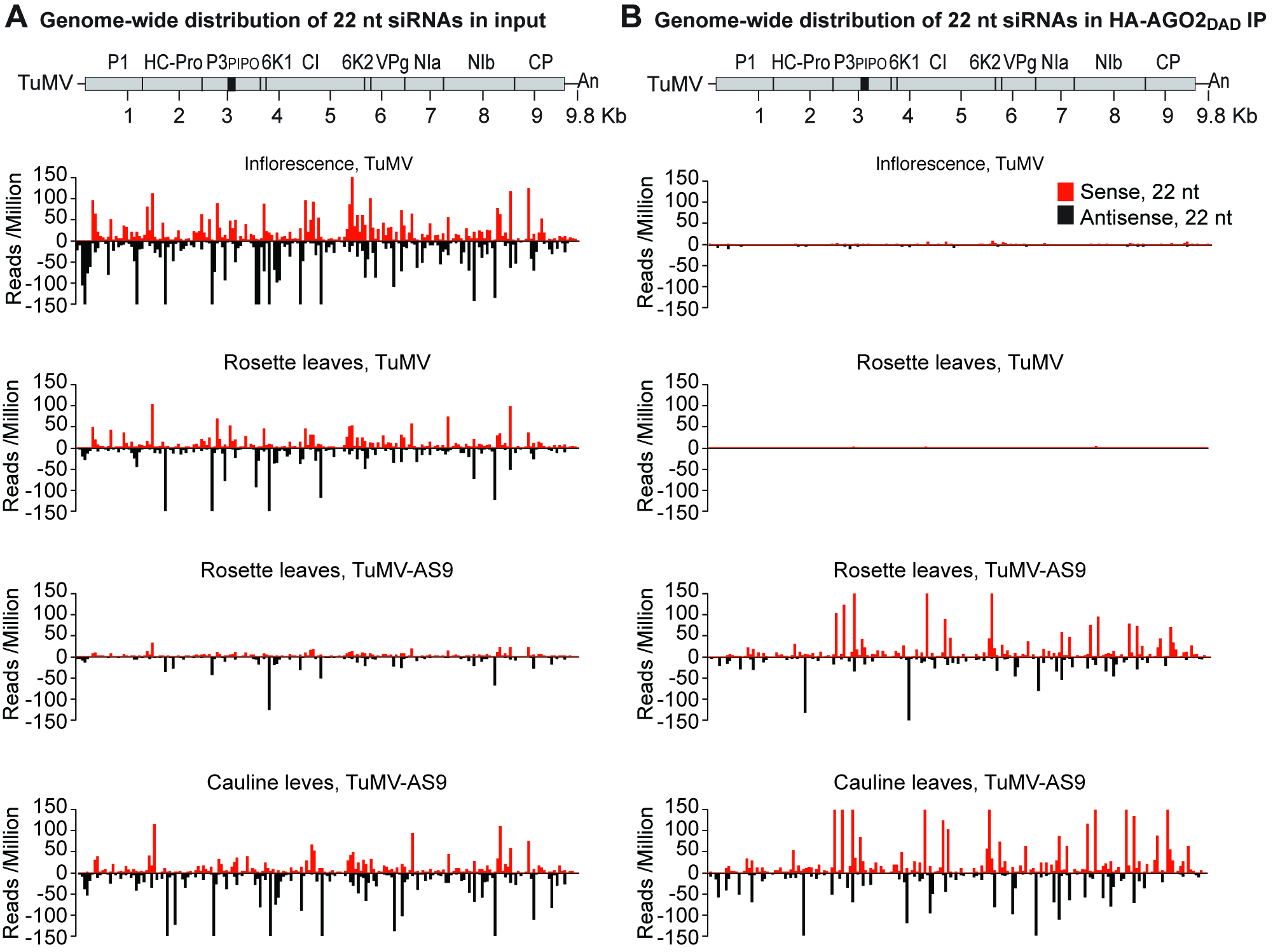

Supplement: S3 Fig — Values are average and SE from two biological replicates normalized to reads per million. Scale was capped at 150. Inoculated rosette leaf and systemically infected cauline leaf samples were collected at 7 and 15 dpi, respectively. Inflorescence samples were collected at 10 dpi. (A) and (B) TuMV genome-wide distribution of 22 nt TuMV-derived siRNAs in input (A) and in HA-AGO2DAD immunoprecipitated (IP) fractions (B). Scale was capped at 150. (TIF) [file ppat.1004755.s003.tif]

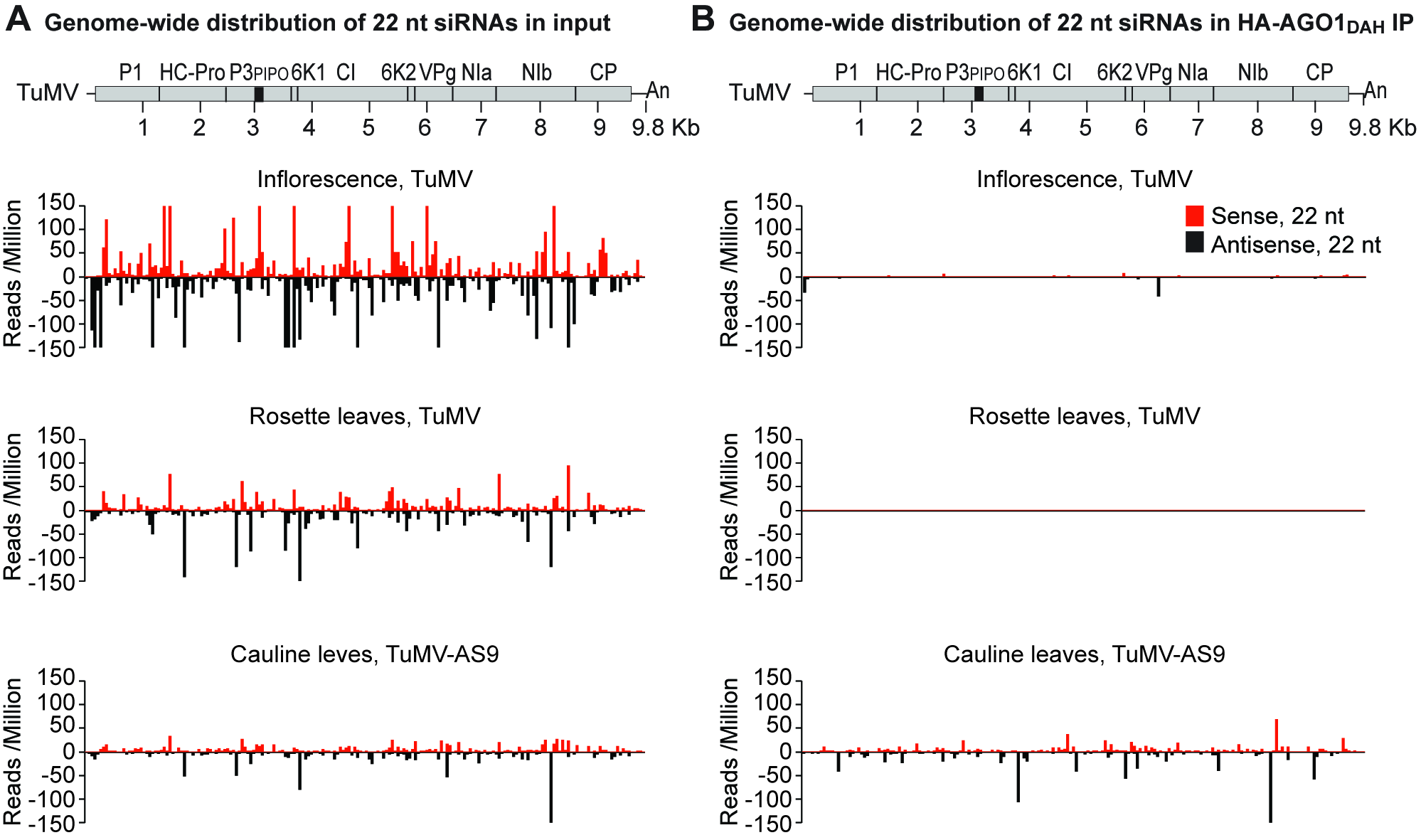

Supplement: S4 Fig — Values are average and SE from two replicates normalized to reads per million. Inflorescence samples were collected at 10 dpi. Inoculated rosette leaf and systemically infected cauline leaf samples were collected at 7 and 15 dpi, respectively. (A) and (B) TuMV genome-wide distribution of 22 nt TuMV-derived siRNAs in input (A) and in HA-AGO1DAH immunoprecipitated fractions (IP) (B). Scale was capped at 150. (TIF) [file ppat.1004755.s004.tif]

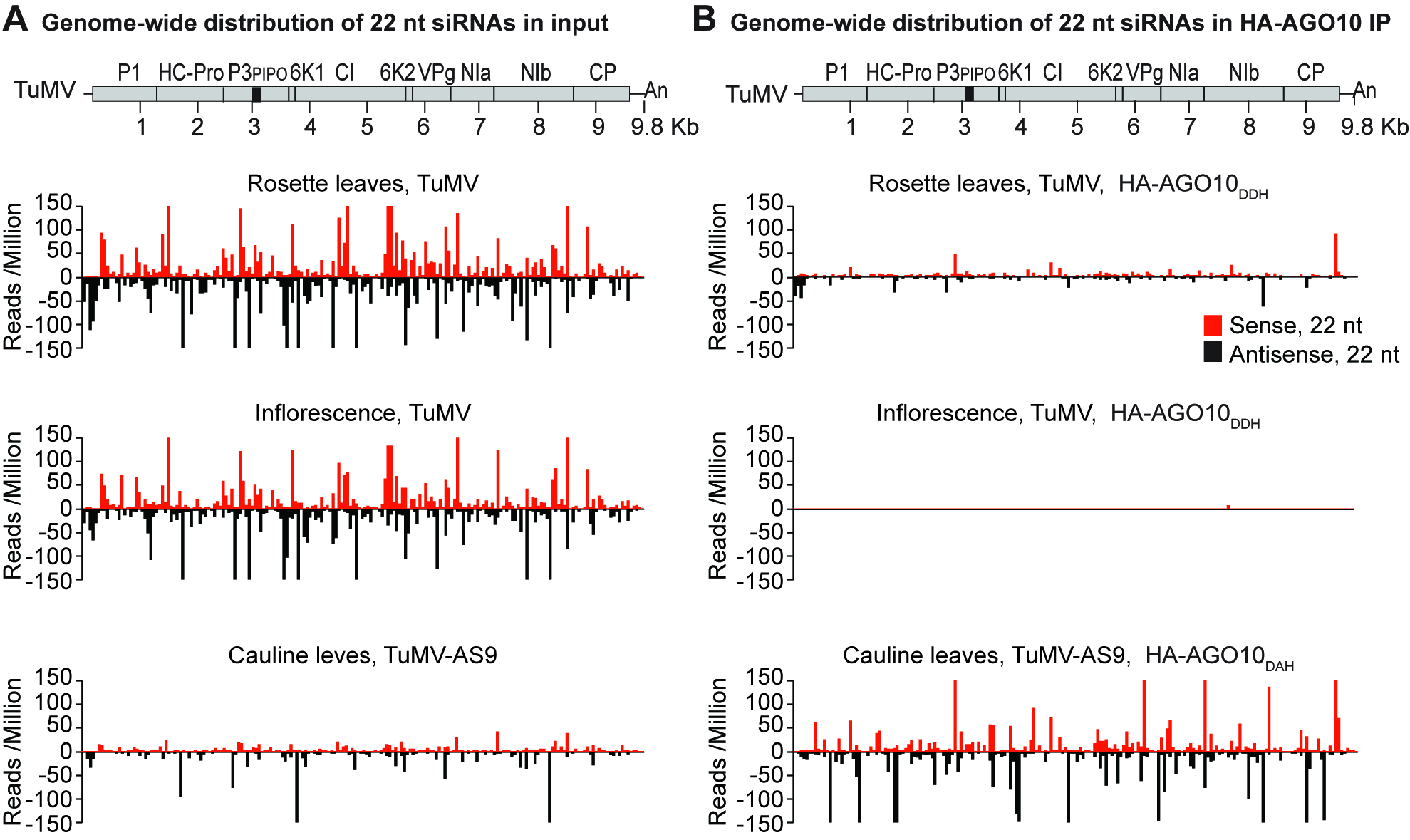

Supplement: S5 Fig — Values are average and SE from two replicates normalized to reads per million. Inflorescence samples were collected at 10 dpi. Inoculated rosette leaf and systemically infected cauline leaf samples were collected at 7 and 15 dpi, respectively. (A) and (B) TuMV genome-wide distribution of 22 nt TuMV-derived siRNAs in input (A) and in HA-AGO10 immunoprecipitated (IP) fractions (B). Scale was capped at 150. (TIF) [file ppat.1004755.s005.tif]

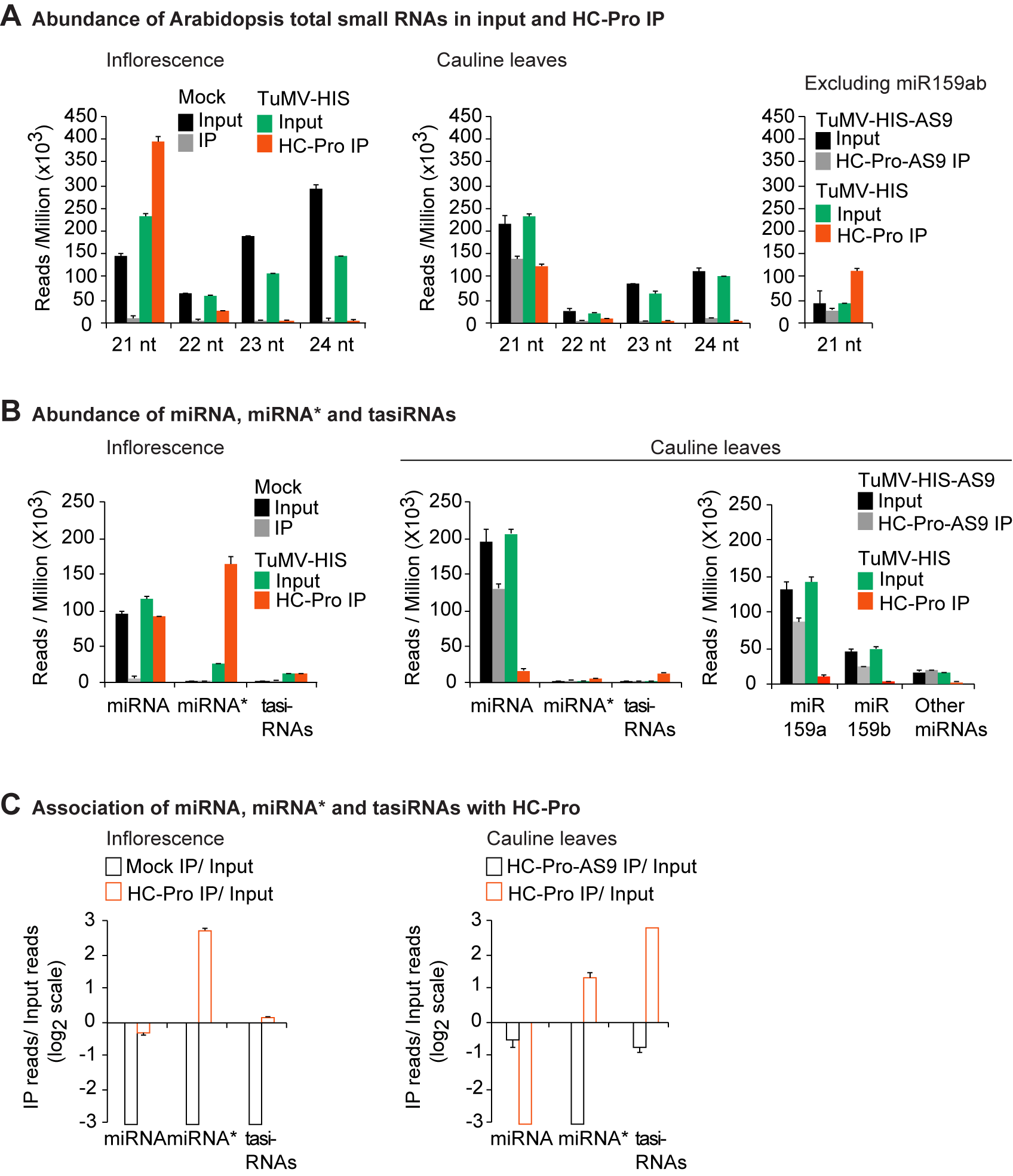

Supplement: S6 Fig — Values are average and SE from two biological replicates normalized to reads per million. Inflorescence and cauline leaf samples from plants infected with TuMV-HIS were collected at 10 dpi. Cauline leaf samples from plants infected with TuMV-HIS-AS9 were collected at 15 dpi. (A) Number of reads of endogenous A. thaliana siRNAs by size class in input and HC-Pro immunoprecipitated (IP) fractions from inflorescence and cauline leaves. (B) Number of reads for miRNAs, miRNA* and tasiRNAs in input and mock or HC-Pro IP. (C) Enrichment (IP reads/ Input reads, expressed in a log2 scale) of miRNAs, miRNA* and tasiRNAs (TAS) in mock or HC-Pro IP. Scales was capped at 3 and -3. (TIF) [file ppat.1004755.s006.tif]

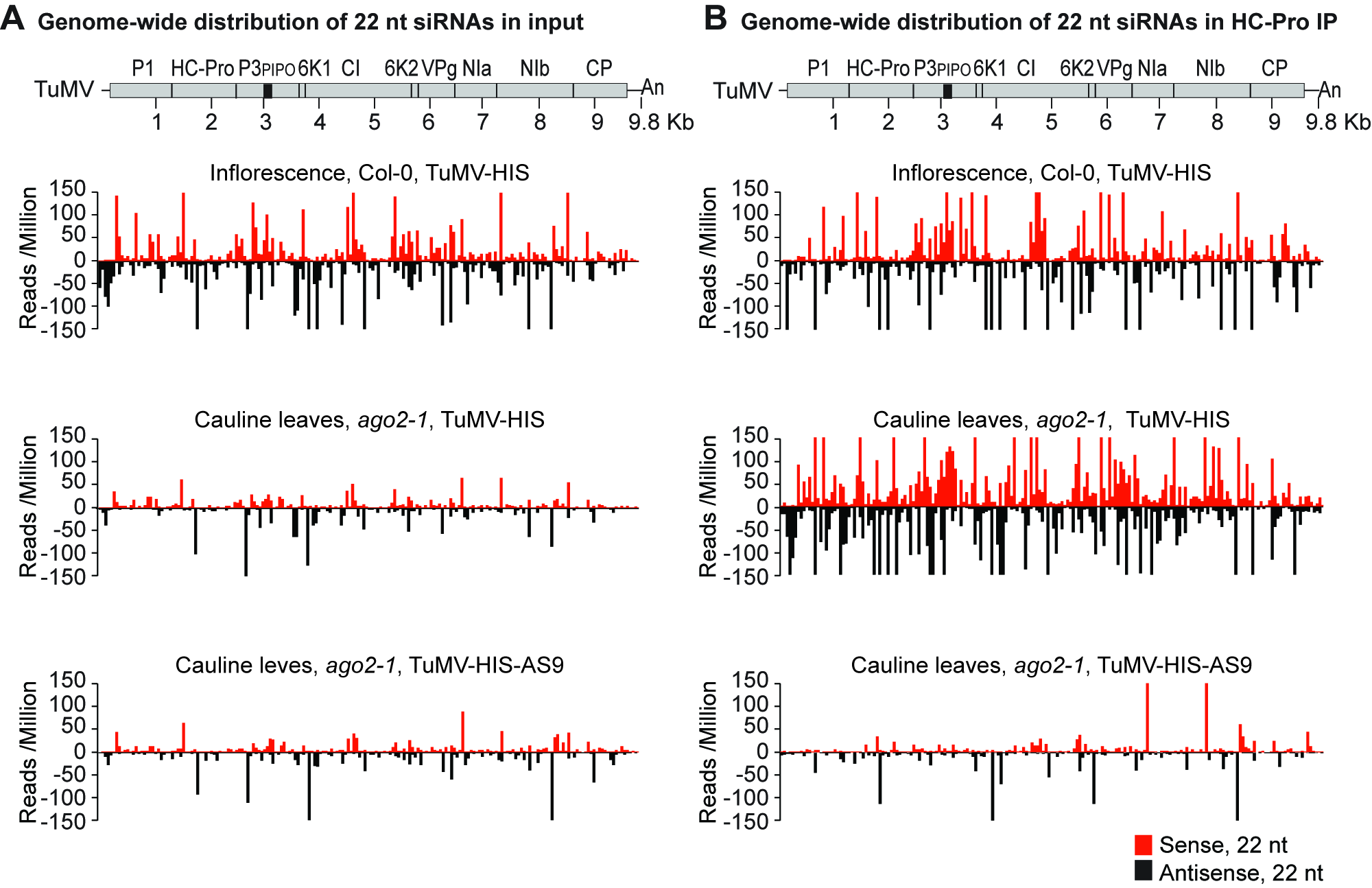

Supplement: S7 Fig — Values are average and SE from two biological replicates normalized to reads per million. Scale was capped at 500. Inflorescence samples were from Col-0 plants at 10 dpi. Cauline leaf samples were from single ago2–1 mutant plants infected with TuMV-HIS or TuMV-HIS-AS9 at 10 or 15 dpi, respectively. (A) and (B) TuMV genome-wide distribution of 22 nt TuMV-derived siRNAs in input (A) or immunoprecipitated (IP) fractions of wild-type or AS9 HC-Pro. (TIF) [file ppat.1004755.s007.tif]

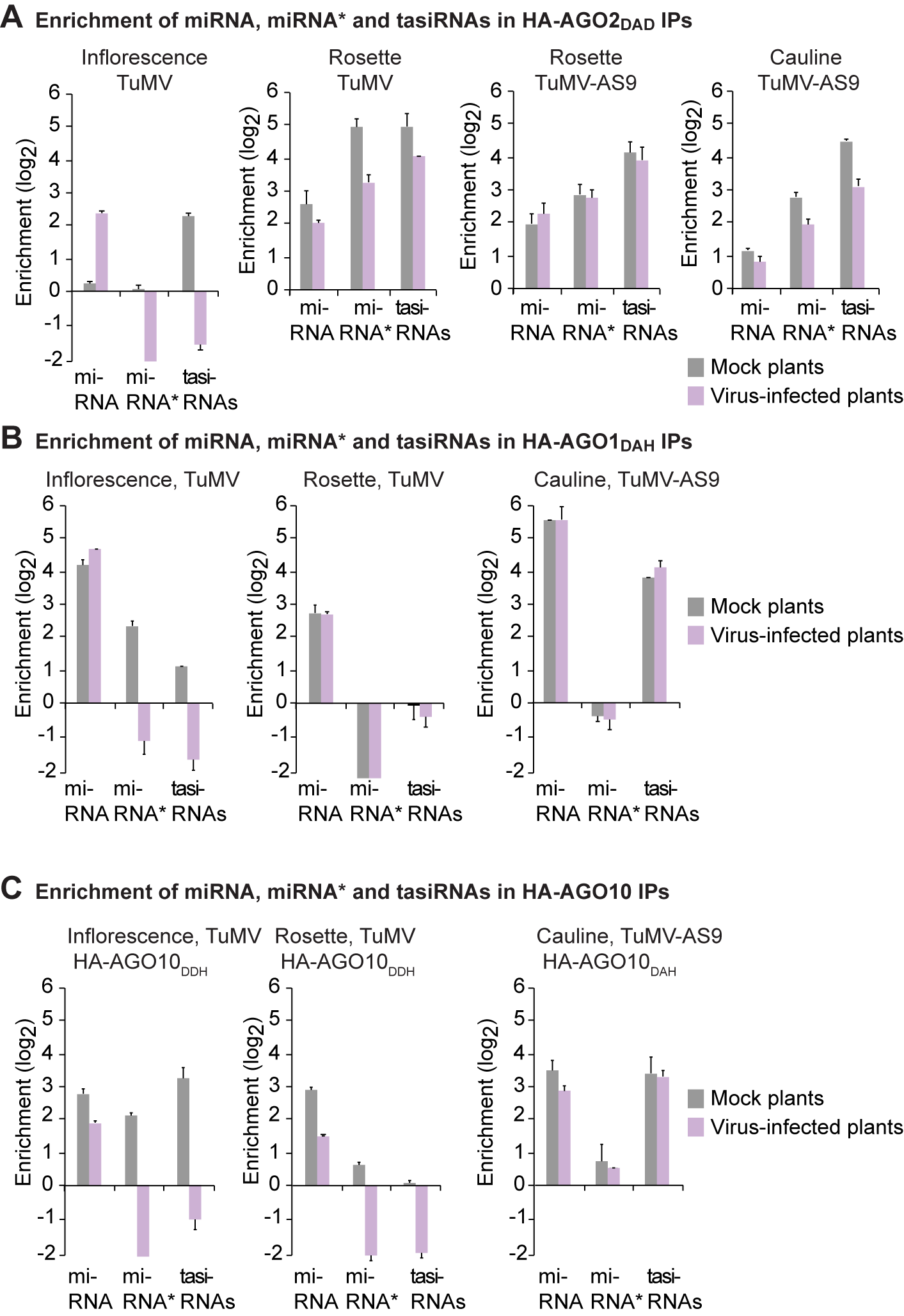

Supplement: S8 Fig — Transgenic HA-AGO1DAH and HA-AGO2 DAD were expressed from an ago2–1 background. Transgenic HA-AGO10DDH and HA-AGO10DAH were expressed from a wild-type Col-0 (AGO2) or an ago2–1 background, respectively. Plants were mock-inoculated or infected with TuMV or with TuMV-AS9. Rosette leaf and samples were collected at 7 dpi. Cauline leaf and inflorescence samples were collected at 15 and 10 dpi, respectively. Values are average and SE from two biological replicates. The histograms show average fold enrichment in AGO IP (IP reads/ input reads, expressed in log2 scale) of miRNAs, miRNA* and tasiRNAs. A) HA-AGO2DAD IP. B) HA-AGO1DAH IP, and C) HA-AGO10DDH or HA-AGO10DAH IP. (TIF) [file ppat.1004755.s008.tif]

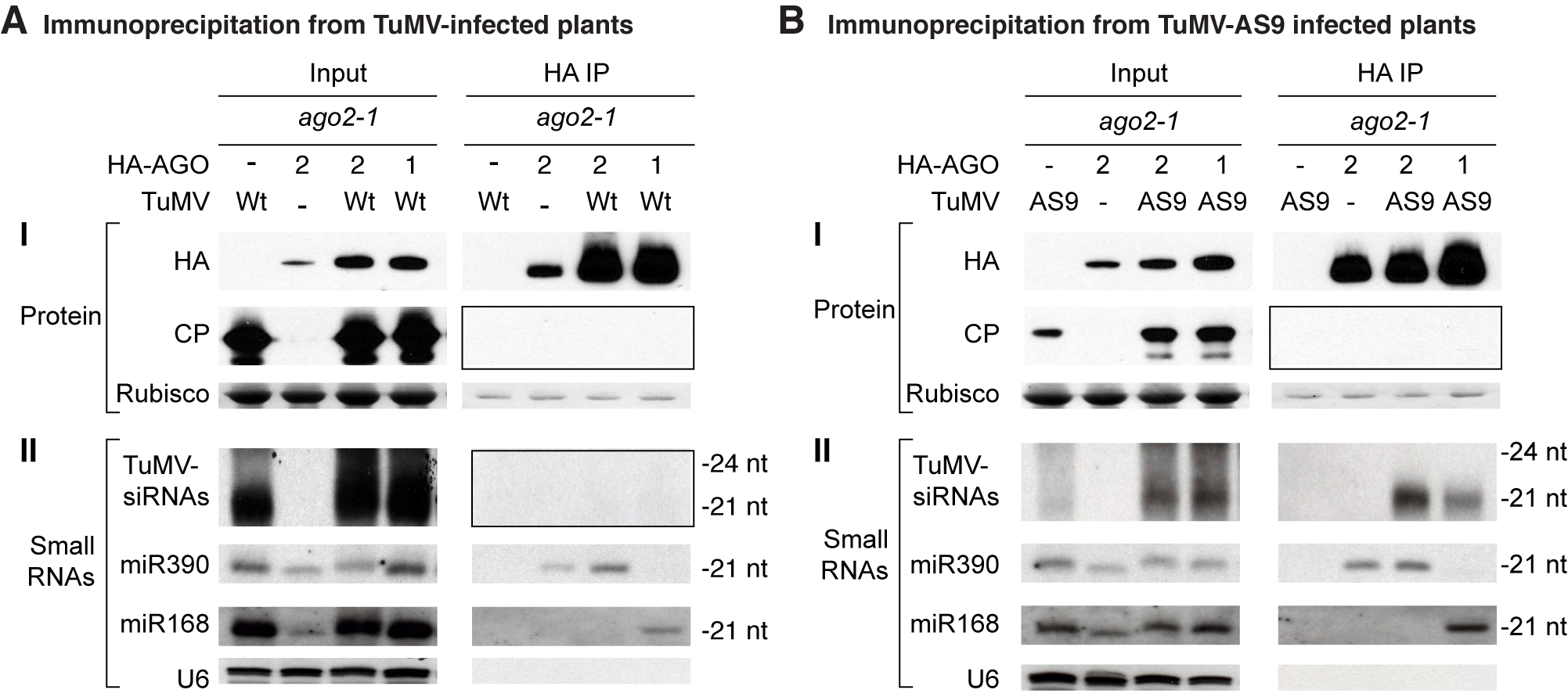

Supplement: S9 Fig — Blots show accumulation of CP, HA-AGO, and virus-derived small RNAs in immunoprecipitation (IP) fractions of HA-AGO1DAH and HA-AGO2DAD from cauline leaves (1g) at 15 dpi. HA-AGO1DAH and HA-AGO2DAD were expressed from transgenic ago2–1 plants. Mock-inoculated plants and non-trangenic single ago2–1 mutants were used as controls. Representative blots showing accumulation of HA-AGOs, CP, TuMV-derived siRNAs (CI) and selected miRNAs in input and HA-AGO immunoprecipitation fractions (IPs). TuMV CP and HA-AGO were detected by immunoblotting in input and IP fractions. TuMV-derived siRNAs were detected with a DIG-labeled probe made by random priming of cDNA corresponding to CI. miR390 and miR168 were used as IP controls, and U6 as loading control. Endogenous siRNAs were detected with DIG-labeled oligonucleotides. Duplicated blots were stripped and re-probed. A) IP of HA-AGO1DAH and HA-AGO2DAD from cauline leaves of plants infected with wt TuMV. Panel I: protein accumulation in input samples. Panel II: protein accumulation in IP fractions. B) IP of HA-AGO1DAH and HA-AGO2DAD from cauline leaves of plants infected with suppressor-deficient TuMV-AS9. Panels I and II are as in (A). (TIF) [file ppat.1004755.s009.tif]
