# Supplementary material for: Roles and Programming of Arabidopsis ARGONAUTE Proteins during Turnip Mosaic Virus Infection
Source: PLoS Pathog. 2015 Mar 25;11(3):e1004755. doi: 10.1371/journal.ppat.1004755 (PMC4373807; doi:10.1371/journal.ppat.1004755)
Supplement: S1 Table — (DOCX) [file ppat.1004755.s010.docx]

**Table S1.** Abundance of endogenous Arabidopsis and TuMV-derived small RNAs (all size classes) in input and HA-AGO2_DAD_ immunoprecipitation fractions ^a^.

| **Genotype** | **Tissue** | **Virus** | **Fraction** | **Total reads**^b^ | **Perfect match**^c^ | **Reads to**  ***Arabidopsis***^d^ | **Reads to TuMV**^d^ |
| --- | --- | --- | --- | --- | --- | --- | --- |
| HA-AGO2_DAD_ | Rosette leaves | Mock | Input | 43,373,442 | 13,076,466  (30%) | 13,075,829  (99.99%) | 638  (0.005%) |
| (*ago2-1*) |  |  | AGO2 IP | 8,545,413 | 7,816,837  (91.5%) | 7,816,790  (99.99%) | 47  (0.001%) |
|  | Rosette leaves | TuMV | Input | 7,705,632 | 3,320,489  (43.1%) | 2,826,369  (85.1%) | 494,120  (14.9%) |
|  |  |  | AGO2 IP | 8,029,687 | 7,548,111  (94%) | 7,542,347  (99.9%) | 5,764  (0.1%) |
| HA-AGO2_DAD_ | Rosette leaves | Mock | Input | 9,775,186 | 4,039,336  (41.3% | 4,039,265  (99.99%) | 71  (0.002%) |
| (*ago2-1*) |  |  | AGO2 IP | 11,704,168 | 10,357,532  (88.5%) | 10,357,297  (99.99%) | 235  (0.002%) |
|  | Rosette leaves | TuMV-AS9 | Input | 16,210,018 | 4,967,191  (30.6%) | 4,660,006  (93.8%) | 307,185  (6.2%) |
|  |  |  | AGO2 IP | 14,647,730 | 12,990,743  (88.7%) | 10,981,824  (84.5%) | 2,008,920  (15.5%) |
| HA-AGO2_DAD_ | Cauline leaves | Mock | Input | 13,729,784 | 6,382,321  (46.5%) | 6,382,259  (99.99%) | 63  (0.001%) |
| (*ago2-1*) |  |  | AGO2 IP | 11,067,283 | 10,086,019  (91.1) | 10,085,901  (99.99%) | 118  (0.001%) |
|  | Cauline leaves | TuMV-AS9 | Input | 12,353,750 | 7,479,449  (60.5%) | 6,425,621  (85.9%) | 1,053,828  (14.1%) |
|  |  |  | AGO2 IP | 16,177,518 | 15,012,694  (92.8%) | 9,469,147  (63.1%) | 5,543,547  (36.9%) |
| HA-AGO2_DAD_ | Inflo-rescence | Mock | Input | 37,857,761 | 30,926,279  (81.7%) | 30,925,036  (99.99%) | 1,243  (0.004) |
| (*ago2-1*) |  |  | AGO2 IP | 38,705,583 | 34,238,610  (88.5) | 34,237,355  (99.99%) | 1,255  (0.004) |
|  | Inflo-rescence | TuMV | Input | 30,427,555 | 23,732,067  (78%) | 19,346,360  (81.5%) | 4,385,707  (18.5% |
|  |  |  | AGO2 IP | 38,823,985 | 31,149,678  (80.2%) | 30,879,035  (99.1%) | 270,643  (0.9%) |

^a^ Read counts are averages of two biological replicates before normalization to reads per million.

^b^ Total number of reads after parsing 5’ and 3’ adaptors.

^c^ Number of reads with a perfect match to Arabidopsis or to TuMV. Numbers in parentheses are relative abundance in percentage of the total reads.

^d^ Numbers in parentheses are relative abundance, in percentage, of reads with a perfect match to Arabidopsis or to TuMV, with respect to the total number of reads with a perfect match.
