# Supplementary material for: Roles and Programming of Arabidopsis ARGONAUTE Proteins during Turnip Mosaic Virus Infection
Source: PLoS Pathog. 2015 Mar 25;11(3):e1004755. doi: 10.1371/journal.ppat.1004755 (PMC4373807; doi:10.1371/journal.ppat.1004755)
Supplement: S2 Table — (DOCX) [file ppat.1004755.s011.docx]

**Table S2.** Abundance of endogenous Arabidopsis and TuMV-derived siRNAs of all size classes in input and AGO1_DAH_ immunoprecipitation fractions^a^.

| **Genotype** | **Tissue** | **Virus** | **Fraction** | **Total reads**^b^ | **Perfect match**^c^ | **Reads to**  ***Arabidopsis***^d^ | **Reads to TuMV**^d^ |
| --- | --- | --- | --- | --- | --- | --- | --- |
| HA-AGO1_DAH_ | Inflo-  rescence | Mock | Input | 23,467,097 | 19,371,562  (82.5%) | 19,370,943  (99.99%) | 619  (0.003%) |
| (Col-0) |  |  | AGO1 IP | 43,435,568 | 39,221,692  (90.3%) | 39,221,677  (99.99%) | 15  (0.0001%) |
|  | Inflo-  rescence | TuMV | Input | 27,870,837 | 24,149,180  (86.6%) | 18,815,740  (77.9%) | 5,333,441  (22.1%) |
|  |  |  | AGO1 IP | 40,247,252 | 36,003,428  (89.5) | 35,956,556  (99.9%) | 46,872  (0.13%) |
| HA-AGO1_DAH_ | Rosette leaves | Mock | Input | 11,343,155 | 7,258,479  (64%) | 7,258,173  (99.99%) | 306  (0.004%) |
| (Col-0) |  |  | AGO1 IP | 17,856,984 | 17,348,967  (97.2%) | 17,348,906  (99.99%) | 61  (0.0001%) |
|  | Rosette leaves | TuMV | Input | 15,115,529 | 7,466,267  (49.4%) | 6,644,115  (89%) | 822,152  (11%) |
|  |  |  | AGO1 IP | 15,920,321 | 15,464,056  (97.1) | 15,463,677  (99.99%) | 379  (0.002%) |
| HA-AGO1_DAH_ | Cauline  leaves | Mock | Input | 11,875,811 | 4,729,312  (39.8%) | 4,728,635  (99.99%) | 677  (0.01%) |
| (*ago2-1*) |  |  | AGO1 IP | 11,547,969 | 9,735,334  (84.3%) | 9,735,018  (99.99%) | 316  (0.003%) |
|  | Cauline leaves | TuMV-AS9 | Input | 10,723,580 | 4,716,647  (44%) | 4,379,171  (92.8%) | 337,476  (7.2%) |
|  |  |  | AGO1 IP | 10,219,912 | 8,548,800  (83.6%) | 8,391,563  (98.1%) | 157,238  (1.8%) |
|  | Cauline leaves | TuMV-AS9 | Input | 12,010,198 | 6,927,457  (57.7%) | 6,049,181  (87.3%) | 878,277  (12.7%) |
|  |  |  | AGO1 IP | 16,958,956 | 14,840,863  (87.5%) | 14,237,830  (95.9%) | 603,033  (4.1%) |

^a^ Values are average of two biological replicates before normalization to reads per million.

^b^ Total number of reads after parsing 5’ and 3’ adaptors.

^c^ Number of reads with a perfect match to Arabidopsis or to TuMV. Numbers in parenthesis are relative abundance in percentage of the total reads.

^d^ Numbers in parenthesis are relative abundance, in percentage, of reads with a perfect match to Arabidopsis or to TuMV, respect to total reads with a perfect match.
