# Supplementary material for: Roles and Programming of Arabidopsis ARGONAUTE Proteins during Turnip Mosaic Virus Infection
Source: PLoS Pathog. 2015 Mar 25;11(3):e1004755. doi: 10.1371/journal.ppat.1004755 (PMC4373807; doi:10.1371/journal.ppat.1004755)
Supplement: S3 Table — (DOCX) [file ppat.1004755.s012.docx]

**Table S3.** Abundance of endogenous Arabidopsis and TuMV-derived siRNAs of all size classes in input and AGO10 immunoprecipitation fractions^a^.

| **Genotype** | **Tissue** | **Virus** | **Fraction** | **Total**  **reads** ^b^ | **Perfect match**^c^ | **Reads to**  **Arabidopsis**^d^ | **Reads to TuMV**^d^ |
| --- | --- | --- | --- | --- | --- | --- | --- |
| HA-AGO10_DDH_ (Col-0) | Rosette leaves | Mock | Input | 11,877,649 | 4,443,886  (37.4%) | 4,442,761 (99.98%) | 1,125  (0.025%) |
|  |  |  | AGO10 IP | 16,253,654 | 14,517,598  (89.3%) | 14,516,733  (99.99%) | 865  (0.006% |
|  | Rosette leaves | TuMV | Input | 18,169,107 | 10,535,498  (58%) | 7,372,836  (70%) | 3,162,662  (30%) |
|  |  |  | AGO10 IP | 16,765,281 | 14,473,800  (86.33%) | 14,456,032  (99.9%) | 17,762  (0.1%) |
| HA-AGO10_DDH_ (Col-0) | Inflo-rescence | Mock | Input | 12,512,734 | 9,752,096  (77.9%) | 9,751,250  (99.99%) | 846  (0.01%) |
|  |  |  | AGO10 IP | 21,133,157 | 18,549,589  (87.8%) | 18,548,697  (99.99%) | 892  (0.005%) |
|  | Inflo-rescence | TuMV | Input | 19,081,851 | 18,890,009  (99%) | 12,588,515  (66.6%) | 6,301,494  (33.4%) |
|  |  |  | AGO10 IP | 11,193,081 | 10,654,979  (95.2%) | 9,884,667  (92.8%) | 770,312  (7.2%) |
| HA-AGO10_DAH_ | Cauline leaves | Mock | Input | 13,726,769 | 3,899,852  (28.4%) | 3,899,676  (99.99%) | 176  (0.005%) |
| (*ago2-1*) |  |  | AGO10 IP | 9,223,666 | 7,768,535  (84.2%) | 7,767,691  (99.99%) | 844  (0.01%) |
|  | Cauline leaves | TuMV-AS9 | Input | 15,549,877 | 5,329,817  (34.3%) | 4,832,266  (90.7%) | 497,551  (9.3%) |
|  |  |  | AGO10 IP | 8,778,803 | 7,152,652  (81.5%) | 6,451,223  (90.2%) | 701,429  (9.8%) |

^a^ Values are average of two biological replicates before normalization to reads per million.

^b^ Total number of reads after parsing 5’ and 3’ adaptors.

^c^ Number of reads with a perfect match to Arabidopsis or to TuMV. Numbers in parenthesis are relative abundance in percentage of the total reads.

^d^ Numbers in parenthesis are relative abundance, in percentage, of reads with a perfect match to Arabidopsis or to TuMV, respect to total reads with a perfect match.
