# Supplementary material for: Roles and Programming of Arabidopsis ARGONAUTE Proteins during Turnip Mosaic Virus Infection
Source: PLoS Pathog. 2015 Mar 25;11(3):e1004755. doi: 10.1371/journal.ppat.1004755 (PMC4373807; doi:10.1371/journal.ppat.1004755)
Supplement: S4 Table — (DOCX) [file ppat.1004755.s013.docx]

**Table S4.** Abundance of endogenous Arabidopsis and TuMV-derived siRNAs of all size classes in input and HC-Pro immunoprecipitation fractions^a^.

| **Genotype** | **Tissue** | **Virus** | **Fraction** | **Total**  **reads**^b^ | **Perfect match**^c^ | **Reads to**  **Arabidopsis**^d^ | **Reads to TuMV**^d^ |
| --- | --- | --- | --- | --- | --- | --- | --- |
| Col-0 | Inflo- rescence | Mock | Input | 10,730,328 | 8,426,808  (78.5%) | 8,426,568  (99.99%) | 240  (0.003%) |
|  |  |  | Mock IP | 24,486 | 18,508  (75.6%) | 18,320  (99%) | 188  (1%) |
|  | Inflo- rescence | TuMV | Input | 15,254,898 | 12,847,479  (84.2%) | 10,455,198  (81.4%) | 2,392,281  (18.6%) |
|  |  |  | HC-Pro IP | 21,699,651 | 19,758,534  (91.1%) | 9,715,707  (49.2%) | 10,042,828  (50.8%) |
| Col-0 | Cauline leaves | Mock | Input | 26,043,288 | 18,085,040  (69.4%) | 18,082,401  (99.99%) | 2,639  (0.01%) |
|  |  |  | Mock IP | 34,124 | 16,479  (48.3%) | 16,122  (97.8%) | 357  (2.2%) |
|  | Cauline leaves | TuMV | Input | 21,939,838 | 9,754,527  (44.5%) | 7,661,360  (78.5%) | 2,093,167  (21.5%) |
|  |  |  | HC-Pro IP | 1,537,090 | 1,439,486  (93.7%) | 214,910  (14.9%) | 1,224,576  (85.1%) |
| *ago2-1* | Cauline leaves | TuMV | Input | 13,713,797 | 7,914,556  (57.7%) | 7,201,376  (91%) | 713,180  (9%) |
|  |  |  | HC-Pro IP | 26,576,212 | 24,947,806  (93.9%) | 3,792,824  (15.2%) | 21,154,982  (84.8%) |
|  | Cauline leaves | TuMV-AS9 | Input | 11,626,228 | 6,964,110  (59.9%) | 6,338,098  (91%) | 626,012  (9%) |
|  |  |  | HC-Pro-AS9 IP | 3,114,131 | 2,268,636  (72.8%) | 2,139,134  (94.3%) | 129,502  (5.7%) |

^a^ Values are average of two biological replicates before normalization to reads per million.

^b^ Total number of reads after parsing 5’ and 3’ adaptors. The average reads per million for the entire flow cell was used as normalization factor for mock-IP and HC-Pro-AS9 IP.

^c^ Number of reads with a perfect match to Arabidopsis or to TuMV. Numbers in parenthesis are relative abundance in percentage of the total reads.

^d^ Numbers in parenthesis are relative abundance, in percentage, of reads with a perfect match to Arabidopsis or to TuMV, respect to total reads with a perfect match.
